# Trophic interactions modify the temperature dependence of community biomass and ecosystem function

**DOI:** 10.1371/journal.pbio.2006806

**Published:** 2019-06-10

**Authors:** Jessica Garzke, Stephanie J. Connor, Ulrich Sommer, Mary I. O’Connor

**Affiliations:** 1 Geomar Helmholtz Centre for Ocean Research Kiel, Department of Experimental Ecology – Food Webs, Germany; 2 Institute of the Oceans and Fisheries, University of British Columbia, Vancouver, Canada; 3 Canadian Rivers Institute, Department of Biology, University of New Brunswick, Fredericton, Canada; 4 Department of Zoology and Biodiversity Research Centre, University of British Columbia, Vancouver, Canada

## Abstract

Aquatic ecosystems worldwide continue to experience unprecedented warming and ecological change. Warming increases metabolic rates of animals, plants, and microbes, accelerating their use of energy and materials, their population growth, and interaction rates. At a much larger biological scale, warming accelerates ecosystem-level processes, elevating fluxes of carbon and oxygen between biota and the atmosphere. Although these general effects of temperature at finer and broader biological scales are widely observed, they can lead to contradictory predictions for how warming affects the structure and function of ecological communities at the intermediate scale of biological organization. We experimentally tested the hypothesis that the presence of predators and their associated species interactions modify the temperature dependence of net ecosystem oxygen production and respiration. We tracked a series of independent freshwater ecosystems (370 L) over 9 weeks, and we found that at higher temperatures, cascading effects of predators on zooplankton prey and algae were stronger than at lower temperatures. When grazing was weak or absent, standing phytoplankton biomass declined by 85%–95% (<1-fold) over the temperature gradient (19–30 °C), and by 3-fold when grazers were present and lacked predators. These temperature-dependent species interactions and consequent community biomass shifts occurred without signs of species loss or community collapse, and only modestly affected the temperature dependence of net ecosystem oxygen fluxes. The exponential increases in net ecosystem oxygen production and consumption were relatively insensitive to differences in trophic interactions among ecosystems. Furthermore, monotonic declines in phytoplankton standing stock suggested no threshold effects of warming across systems. We conclude that local changes in community structure, including temperature-dependent trophic cascades, may be compatible with prevailing and predictable effects of temperature on ecosystem functions related to fundamental effects of temperature on metabolism.

## Introduction

Temperature affects metabolic rates of all organisms, thereby affecting ecological patterns and processes across scales of organization—from individuals to ecosystems. Increasing temperature accelerates major metabolic processes that drive net ecosystem production (NEP) and ecosystem respiration (ER) in aquatic and terrestrial ecosystems [1–4]. Highly conserved metabolic processes—photosynthesis and aerobic respiration [5]—power somatic growth, maintenance, and activity in aerobic organisms. As a result, the effects of temperature on cellular photosynthesis and respiration have accurately described the exponential increases in ecosystem-scale ecosystem productivity (NEP) and respiration (ER) in aquatic systems across macroecological thermal gradients, after accounting for body size, nutrient content, and light availability [4,6,7]. The ecological importance of temperature-dependent per capita metabolic rates has supported the use of metabolic models to understand and predict ecological change from local to global scales [3,4,8]. This has been a general theme in the metabolic theory of ecology (MTE). Models that associate change in ecosystem-scale metabolism (e.g., oxygen or carbon flux) with individual-level oxygen production and respiration, but bypass the complexity of population and community dynamics at intermediate biological scales, provide much-needed predictability for how climate change affects ecosystem functions when ecosystems are compared across broad spatial or temporal thermal gradients [2,4,9].

Reconciling the high explanatory power of general temperature-dependent metabolic scaling models at macroecological scales with the well-documented contingencies of how temperature affects community-level outcomes of population dynamics and species interactions at intermediate scales has been challenging [10–17]. Whether at macroecological or community (e.g., single-site) scales, ecosystem-level functions (ER, NEP) or standing stock is simply the sum of per capita function (respiration, net photosynthesis) and biomass. Metabolic theory models applied at macroecological scales assume that the relationship between temperature and community-level distributions of body sizes and traits is constant in time, or that communities are at a stable state so that descriptions of community structure apply to future states of the community under the same abiotic conditions [10–12]. Yet, at local scales, species interactions can influence biomass of primary producers, and the strength and outcomes of species interactions reflect dynamical processes that are often sensitive to temperature [13–15]. For example, the presence of fish in experimental aquatic ponds reversed a negative effect of temperature on algal biomass to a positive effect, mediated by trophic interactions between fish, zooplankton, and phytoplankton [16], under otherwise constant consistent abiotic conditions across ponds. Understanding how temperature-dependent species interactions affect biomass, size distributions, and traits remains a challenge. For example, why do temperature-dependent species interactions influence the effects of temperature on community properties such as biomass, abundance, and body size but have little or no apparent effect on the variation in ecosystem functions (NEP, ER) over macroecological scales? This challenge is central to efforts to apply general models of metabolic temperature dependence to communities [17,18]. This paradox between macroecological patterns—which can be consistent with direct scaling of per capita thermal responses—and results of smaller-scale, short-term experiments that allow population dynamics to play out over intermediate timescales, leads to the suggestion that general metabolic scaling models that do not consider the complexities associated with species interactions do not apply at the local scales [19]. Reconciling these apparently divergent patterns is critical to improving understanding and projections of how shifting global thermal regimes affect ecological patterns and processes across scales and achieving a more unified understanding of ecology across scales.

**Fig 1:**
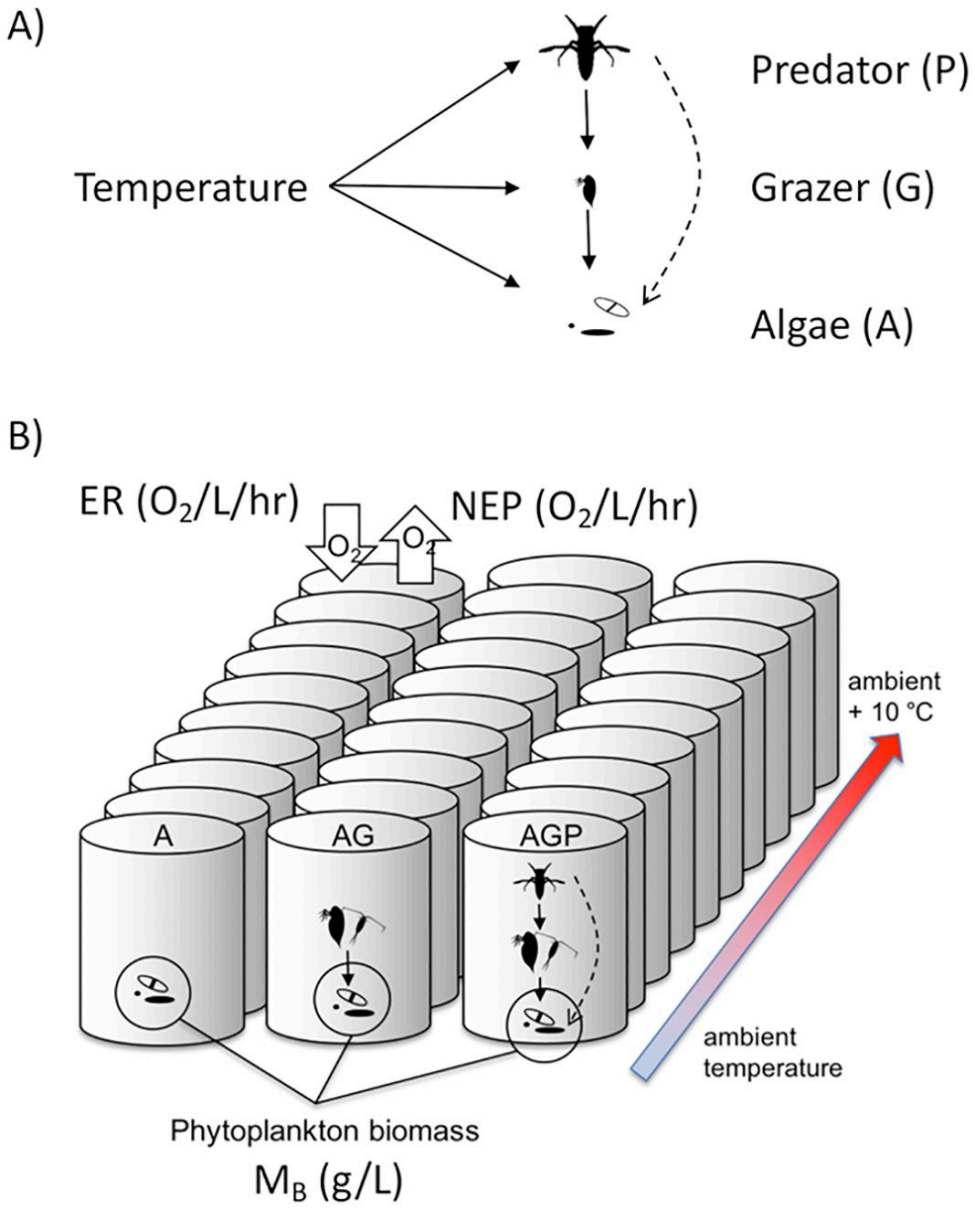
(A) Temperature and predation directly and indirectly affect population density and metabolic rates in aquatic communities. In our experimental communities, predation directly (solid lines) affects the abundance, size, and species composition of prey, and predation by notonectids on grazers leads to an indirect effect (dashed line) called a trophic cascade on algal abundance. Temperature directly affects per capita metabolic rates (solid lines) and indirectly affects algal abundance (dashed lines) by increasing grazing rates, and may have stronger effects on heterotrophic metabolic rates relative to algal metabolic rates (thicker lines represent a stronger direct effect of temperature). Other indirect effects of temperature are possible. (B) Experimental communities varied in their trophic structure. Ten communities included algae only (A), 10 comprised algae + grazers (AG), and 10 included algae + grazers + predators (AGP). We sampled net ecosystem oxygen production (NEP), ecosystem respiration (ER), and total phytoplankton biomass (M_B_) weekly for 8 weeks. AG, algae + grazers; AGP, algae + grazers + predators; ER, ecosystem respiration; M_B_, total phytoplankton biomass; NEP, net ecosystem oxygen production.

One way to reconcile the apparent context dependence of empirical results under controlled conditions with the generality of temperature dependence of ecosystem function at broader scales is to consider how the direct and indirect effects of temperature on population dynamics interact. Direct effects of temperature on per capita metabolic rates cause organismal photosynthesis and respiration rates to increase exponentially when temperatures increase, as long as resources are not limiting in algae and animals, up to an optimal temperature. This relationship between temperature and fundamental metabolic rates (photosynthesis and respiration) is referred to as general metabolic scaling [1]. For any single phenotype, performance above some optimal temperature declines due to stress responses and metabolic scaling no longer explains the effects of temperature on performance. However, in multispecies communities, the signal of metabolic scaling is likely to dominate over a broad range of temperatures if species with distinct thermal phenotypes can compensate for each other along the thermal gradient [10,20]. Warming is also associated with other biological changes that affect species interactions, such as reductions in body size (the temperature size rule [21–23]), fecundity, and attack rates (Fig 1) [24–27], and these changes can feed back to influence community-level biomass and productivity [28–30].

The temperature dependence of consumer-resource interactions—mediated by dynamics of two or more populations—has received substantial attention in this context, because these trophic interactions can influence many aspects of community structure and ecosystem function, including biomass, abundance, species composition, and stability [10,12,31,32,34]. Trophic species interactions appear to strengthen with warming [16,29,31]. Series of trophic interactions, called trophic cascades (Fig 1), link predator populations to the abundance, biomass, and ecosystem functions of primary producers [32,33]. The strength of trophic cascades depends on predator and prey body sizes and primary production [34]. Considering the prevalence of consumer-resource interactions and trophic cascades in aquatic systems [35] begs the question, how is it that population-level responses to temperature [28,36] do not appear to cause major variation or context dependence in macroecological relationships between subcellular metabolic processes (photosynthesis, respiration) and ecosystem processes (NEP, ER)?

Here, we aimed to resolve the paradox between apparent direct effects of temperature on ecosystem functions (NEP, ER) that emerge when comparing communities across larger gradients and the potentially more complex effects of temperature at the population and community scales. We experimentally tested the hypothesis that temperature-dependent trophic interactions in a trophic cascade alter the effect of temperature on community properties such as biomass, abundance, and body size, but have little or no effect on the variation in ecosystem functions (NEP, ER) over a temperature gradient. In freshwater plankton communities, we compared the effects of temperature on community properties typically measured in warming experiments (e.g., biomass, density, body size) with the effects of temperature typically measured in macroecological studies (e.g., NEP, ER). We controlled variation in biotic and abiotic conditions other than temperature and trophic structure (presence of grazers and predators) (Fig 1). We quantified ecosystem function (NEP, ER) and community structure (biomass, abundance) in ecosystems with algae only (A), algae and grazers (AG), or algae, grazers, and predators (AGP) across an experimental temperature gradient of 10 °C. We found that exponential effects of temperature on algal biomass were greater than effects of temperature on NEP and ER, suggesting that even large changes in community structure do not necessarily lead to large changes in how temperature affects NEP and ER.

## Hypotheses

We drew on the MTE to frame our hypotheses and predictions for how temperature affects NEP and ER via per capita metabolic temperature dependence and indirect effects of temperature at the community scale. We first briefly outline the framework and then express our specific hypotheses. MTE relates whole-organism metabolic rates (*b*_*i*_, gO_2_/hour) and related biological functions for organism *i* to body size (*m*_*i*_, g) and body temperature (*T*, in Kelvin) [1,37,38]:

$$b_{i}=b_{0}e^{-E_{a}/kT}m_{i}^{a}$$ (1)

in which activation energy (*E*_a_, in eV) captures the exponential effect of temperature on per capita metabolic rate, *k* is the Boltzmann constant (eV/K), and *b*_*0*_ is a normalization constant independent of body size and temperature that includes the effects of temperature-independent traits on metabolic rate (gO_2_/g^α^/hour). The allometric scaling factor *α* relates metabolic rate to body size.

The effects of temperature on ecosystem metabolic rates (*B*_*R*_), such as NEP or ER (gO_2_/hour), reflect the sum of all per capita photosynthesis rates by autotrophs and respiration rates by autotrophs and heterotrophs, as well as shifts in abundance, body size, and acclimation. These models implicitly assume an ample and constant supply of resources. Note that NEP and ER can be quantified in this way as positive numbers, and we do this—using their absolute values—in our analyses. Following Barneche and colleagues [10], we capture direct and indirect effects of temperature on ecosystem-scale metabolic rates in the following equation (see Barneche and colleagues [10] for derivation):

BR=b0(TC)e−ER(1kT−1kTC)MB〈mBα−1〉. (2)

The term e−ER(1kT−1kTC) captures the temperature dependence *E*_*R*_ (eV) of ecosystem-level metabolic rate *B*_*R*_. Eq 2 represents a “first-order metabolic scaling” prediction that ecosystem-scale mass-normalized metabolic rates (e.g., NEP) vary proportionally with the temperature dependence of the underlying metabolic processes (e.g., photosynthesis). Observed temperatures, *T*, are related to an arbitrarily chosen reference temperature, *T*_*c*_. This centering causes the normalization constant *b*_*0*_*(T*_*C*_*)* to be for metabolic performance at temperature *T*_*c*_.

When considering the indirect effects of temperature on ecosystem oxygen production and respiration, we can consider how each term in Eq 2 may vary with temperature. To account for changes in total biomass, body size, or relative abundance of phenotypes (traits) associated with temperature, we use the term $M_{B}{\langle m_{i}^{\alpha -1}\rangle }_{B}$. The total biomass, *M*_*B*_ (g), in ecosystem volume V, equals the sum of mass *m*_*i*_ for all individuals *i* to *J* $(M_{B}=\frac{1}{V}\sum_{i=1}^{J}m_{i})$. The term $\langle m_{i}^{\alpha -1}\rangle$ is the average of all individual metabolic biomasses, $\langle m_{i}^{\alpha -1}\rangle =(\sum_{i=1}^{J}m_{i}^{\alpha })/(\sum_{i=1}^{J}m_{i})$, corrected for the greater contribution to total mass-specific metabolic biomass by small individuals resulting from the allometric scaling (*α*)of oxygen production and consumption with body size [2,10]. This “mass correction” is necessary, because if community biomass is comprised of one large individual, that biomass will [produce and] consume less oxygen per gram biomass in a given time period than if the same total biomass were comprised of many small individuals; in other words, $M_{B}\langle m_{B}^{\alpha -1}\rangle$ increases with *m*_*i*_. If thermal traits acclimate or species composition shifts with temperature, this term would capture that change. Therefore, Eq 2 can capture direct effects of temperature on community metabolism via changes in per capita metabolic rate (*E*_*R*_) and via changes in biomass, size distribution, and phenotypes.

### Hypothesis 1: The relationship between algal biomass and temperature is modified by the number of trophic levels

Via strong trophic interactions, predators can change the standing biomass of primary producers in communities. Total algal biomass (*M*_*B*_) can be expressed in terms of temperature, traits, and size distributions:

MB=BReER(1kT−1kTC)b0(TC)〈mBα−1〉. (3)

If we assume that $B_{R}*(b_{0}(T_{C})*{\langle m_{B}^{\alpha -1}\rangle )}^{-1}$ is independent of temperature, we predict that algal biomass *M*_*B*_ declines with temperature by *E*_*R*_; in this case, *E*_*R*_ = −*E*_*NEP*_. This prediction has been supported empirically in a single species algae system [39], and in that system the predicted decline in total biomass was robust to changes in cell size. However, it is unlikely that grazers and temperature would not alter the abundance and size of algae, altering $\langle m_{B}^{\alpha -1}\rangle$ among trophic treatments [40] and also the traits of algae, and thereby modifying *b*_0_ (*T*_*C*_) among trophic treatments [41]. A fuller integration of how temperature and trophic treatment affect these terms for multispecies assemblages would require theoretical development that is beyond the scope of this paper, but we use the equation here to highlight why we expect trophic structure and temperature to affect algal biomass. To test this hypothesis and the alternative, that $B_{R}*(b_{0}(T_{C})*{\langle m_{B}^{\alpha -1}\rangle )}^{-1}$ is independent of temperature, we linearized Eq 3 for analysis by log transforming (Methods, Eq 8), and then we compared ln(*M*_*B*_) trends with temperature across ecosystems with and without a trophic cascade (AGP versus AG ecosystems).

### Hypothesis 2: Increasing temperature strengthens the trophic cascade

We estimated the strength of the trophic cascade as the log ratio of primary producer biomass in the presence of predators (AGP) versus in predator-free environments (AG) [42]. We predicted that predators would reduce the abundance of zooplankton through predation and shift zooplankton composition to smaller sizes and less edible species, typical of classic freshwater trophic cascades [43]. We also predicted that these trophic interactions would strengthen with higher temperatures due to the effect of temperature on per capita zooplankton grazing rates. We can relate algal biomass among treatments using Eq 3 for primary producer biomass in the presence of predators (AGP) and grazers only (AG), simplifying and taking the natural log to yield (see Methods, Eqs 8–11, for details)

$$ln\;(\frac{M_{B.AGP}}{M_{B.AG}})=\frac{\text{ln}\left({b_{0}(T_{C})}_{AG}\right)+ln({\langle m_{B}^{\alpha -1}\rangle }_{AG})-\frac{{E_{b.ag}-E}_{m.ag}}{kT}}{\text{ln}\left({b_{0}(T_{C})}_{AGP}\right)+ln({\langle m_{B}^{\alpha -1}\rangle }_{AGP})-\frac{{E_{b.agp}-E}_{m.agp}}{kT}}$$ (4)

Numerous experiments have demonstrated that the strength of the trophic cascade (log $\frac{M_{B.AGP}}{M_{B.AG}}$) increases with an increase in temperature of a few degrees [29,44,45], and theoretical work suggests that strengthening of this interaction under warming is expected for a greater range of consumer-resource parameter values than would predict declines in the trophic cascade [15]. We therefore predict the trophic cascade strength will increase with temperature in our experiment. But as shown in Eq 4, because trophic interactions could affect the realized temperature dependence via several possible mechanisms—shifts in body size, traits, etc.—it is not possible to predict a priori the temperature dependence of the trophic cascade, in terms of the differences and ratios of the temperature dependence terms in Eq 4 (see Methods: Model and hypothesis development). A “first-order” metabolic scaling prediction would be no change in trophic cascade strength, because the model would assume that the temperature dependences of mass and the normalization constant (*E*_*b*.*ag*_, *E*_*b*.*agp*_, *E*_*m*.*ag*_, and *E*_*m*.*agp*_) all equal 0. We expected the indirect effects of predators on algae to be mediated by changes in zooplankton density and/or body size. Zooplankton attributes are not explicitly modeled in Eq 4 but could contribute to temperature dependence of algal cell size and trait distributions. Reduced zooplankton size or density in the presence of predators could lead to different indirect effects of temperature on algal cell size and traits in the presence versus absence of predators. We tested the prediction that temperature dependence of zooplankton size and density are different from zero (Methods: Statistical analysis).

### Hypothesis 3: Temperature dependence of NEP and respiration depends on the strength of the trophic cascade

We test this by using Eq 2 to model ecosystem-scale NEP and ER, but we allow *b*_*o*_(*T*_*C*_) to vary not only with temperature but with trophic structure (*Z*_*j*_). We expect that trophic structure will influence the number and size of individuals, and thereby affect $M_{B}\langle m_{B}^{\alpha -1}\rangle$. Alternatively, trophic structure may not modify the relationship between temperature and *B*_*R*_, if *b*_*o*_(*T*_*C*_) and $\langle m_{B}^{\alpha -1}\rangle$ are independent of temperature. We can test these alternate predictions by comparing models with and without *b*_*o*_(*T*_*C*_) and $\langle m_{B}^{\alpha -1}\rangle$ terms that depend on ecosystem temperature and trophic structure.

For each hypothesis, we used linear mixed effects models (LMMs) to test “first-order” metabolic scaling models for the appropriate model (Eqs 2, 3, or 4) that included *b*_*o*_(*T*_*C*_) as independent of the ecosystem’s temperature or trophic structure (Methods: Statistical analyses). We tested alternate models that allowed *b*_*o*_(*T*_*C*_) to vary with ecosystem temperature and/or trophic structure. If the simpler, first-order models are best supported, we would infer that indirect effects of temperature do not overwhelm the signals of direct metabolic scaling effects on ecosystem functions, consistent with inferences drawn in macroecological studies. To estimate intercepts and temperature dependence terms (e.g., *E*_*R*_), we summed coefficient values and estimated uncertainties in these aggregated parameters from best models (Methods: Statistical analysis). For each hypothesis, we tested two measures of ecosystem temperature: mean temperature over the 9-week experiment, which captures differences among systems, or weekly mean temperature, which captures differences within ecosystems over time. Our data do not permit testing specific predictions about size distributions or trait shifts, but support for models with variation in *b*_*o*_(*T*_*C*_) and $\langle m_{B}^{\alpha -1}\rangle$ among treatments would suggest these mechanisms as likely explanations.

## Results

### Hypothesis 1

**Fig 2:**
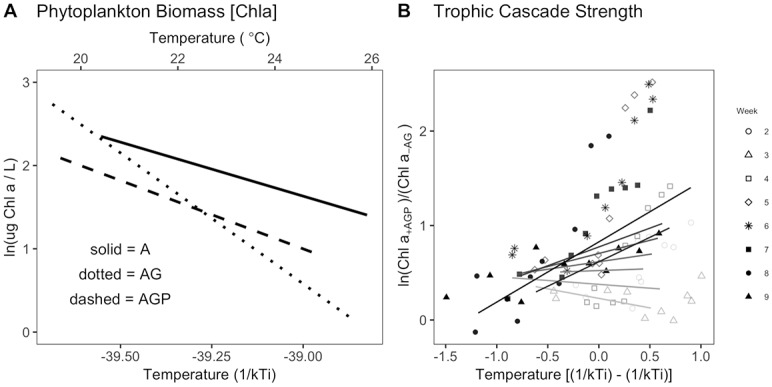
(**A**) Estimated phytoplankton biomass (chlorophyll *a* concentration) declined with increasing temperature and varied with trophic structure (A, AG, AGP). Lines are estimated effects of temperature on phytoplankton biomass based on LMMs (Eq 12) for Eq 3, with temperature dependence in model terms for the intercept and slope (Table 1). From the best model, the intercept and slope of each line were estimated by pooling terms for the intercept and temperature dependence in Eq 12 (see Methods, Eq 14). All observations for phytoplankton biomass are shown in Fig 6. (**B**) Strength of the trophic cascade at a given temperature was estimated by taking the log ratio of algal biomass (estimated as chlorophyll *a* concentration) in the presence of predators and grazers (AGP) versus the algal abundance in the presence of grazers only (AG) (Eq 4, Table 2). Lines represent fixed effects of temperature from the full model (Table 2), centered on the grand mean of all recorded ecosystem temperatures (Eq 13). Gray shading and symbols indicate the week, from week 2 (July 10) to week 9 (August 28), 2012. Data for these figures may be found at https://doi.org/10.5281/zenodo.2652579 in GarzkeAllwks.csv. A, algae only; AG, algae and grazers; AGP, algae, grazers, and predators; Chla, chlorophyll a; LMM, linear mixed effects model.

**Fig 3:**
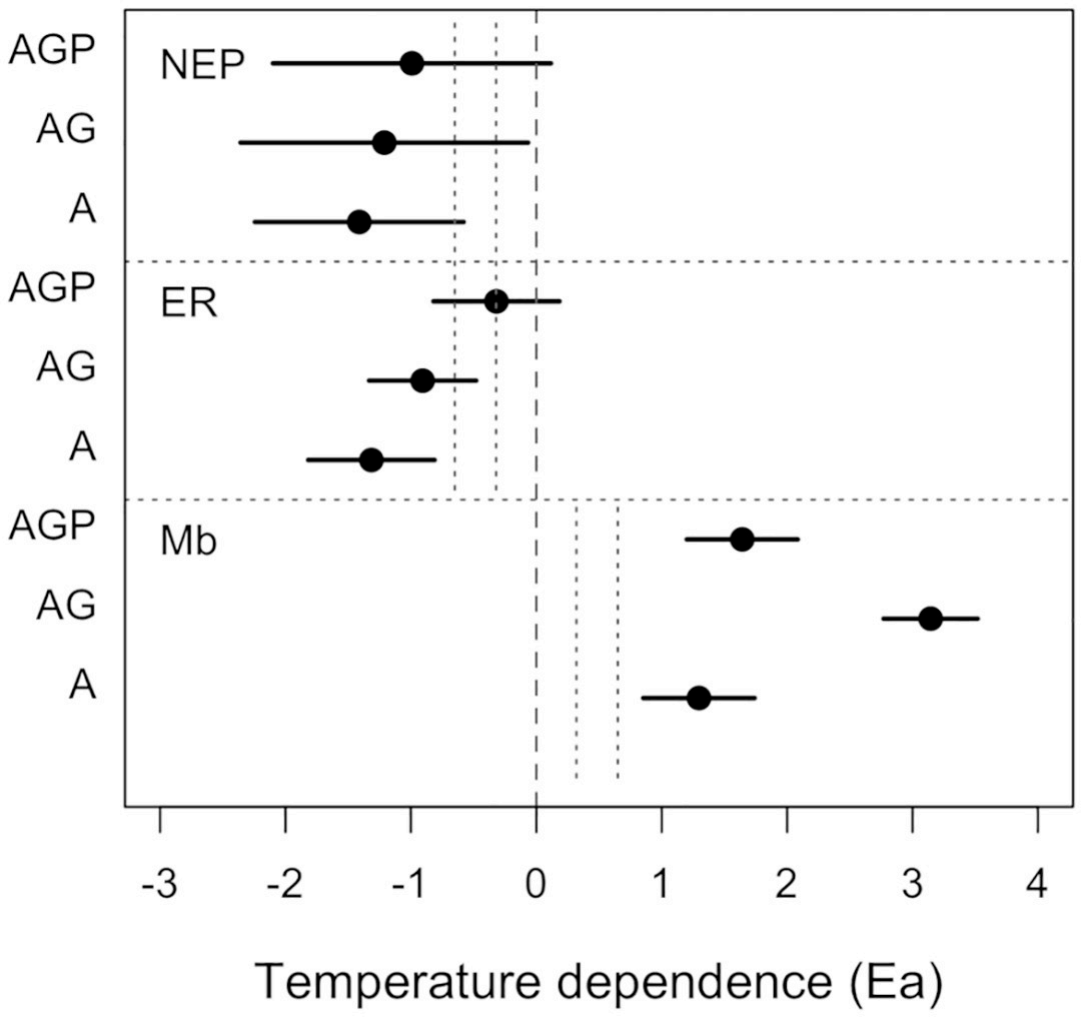
Comparison of estimated temperature dependences of phytoplankton biomass (M_B_), NEP, and ER for communities with algae only (A), algae and grazers (AG), and algae, grazers, and predators (AGP). Composite estimates of temperature dependences are as shown in Figs 2A and 5 (following Methods, Eq 14). No temperature dependence is indicated by the dashed line, and the vertical gray dotted lines indicate 0.65 and 0.32 eV, expected temperature dependences of algal photosynthesis and respiration, and −0.65 and −0.32 as expectations for the temperature dependence of phytoplankton total biomass. Data for these figures may be found at https://doi.org/10.5281/zenodo.2652579 in GarzkeAllwks.csv. A, algae only; AG, algae and grazers; AGP, algae, grazers, and predators; ER, ecosystem respiration; NEP, net ecosystem production.

As temperature increased across ecosystems, phytoplankton biomass, estimated as the concentration of chlorophyll *a* in the water column, declined (Fig 2A). Trophic interactions modified the effect of temperature on chlorophyll *a* concentration (Fig 2A, Table 1). This inference is supported by the inclusion of a main effect for trophic structure (Z_j_) in the best model (Table 1) and an estimate for the temperature dependence of chlorophyll *a* concentration with confidence intervals that exclude 0 (Fig 3). Phytoplankton biomass declined much more strongly with temperature in algae-grazer (predator-free) communities, with a decline of over three orders of magnitude in phytoplankton biomass standing stock over the 10 °C temperature gradient (Fig 2A). In the algae-grazer-predator treatments, phytoplankton biomass declined with a slope indistinguishable from that in the algae-only treatments (Fig 3). We did not observe shifts in taxonomic composition with temperature (S1 Fig).

**Table 1:** Model selection results for LMMs of phytoplankton biomass. The full statistical model (Methods, Eq 12) related ln(chlorophyll *a*) to ecosystem trophic structure (*Z*_*j*_) and average ecosystem temperature over the entire experimental period (*T*_*M*_), while accounting for effects of temperature variation over time (weekly average temperature [T_wj_]) and with ecosystem identity as a random effect. We compared models using likelihood ratios (LogLik), AIC_C_, Akaike weights (w), and δAIC_C_ weights. The model was fit to 240 observations in 30 groups. The full model (PBF) includes all terms, and models representing alternate hypotheses excluded terms indicated by “NA.” Values indicate model-estimated coefficients. Coefficients were pooled (Methods: Statistical analysis) to estimate slopes and intercepts for Figs 2 and 3.

Mod	Int	Z_j_	Twj	T_M_	T_wj_*Z_j_	T_M_*Z_j_	T_M_*T_wj_	df	logLik	AIC_C_	δAIC_C_	w
PBF	2.05	+	−0.52	1.30	1.34	+	+	12	−155.37	336.11	0.00	1.00
PB8	2.05	+	−0.66	1.30	NA	+	+	11	−162.86	348.87	12.76	0.00
PB7	2.05	+	−0.96	1.30	NA	NA	+	9	−168.05	354.89	18.78	0.0
PB4	1.50	NA	−0.96	1.70	0.96	NA	NA	6	−207.94	428.24	92.13	0.0
PB6	1.91	+	−0.66	NA	NA	+	NA	8	−206.58	429.79	93.68	0.0
PB3	1.50	NA	−0.96	1.71	NA	NA	NA	5	−211.73	433.72	97.62	0.0
PB5	1.91	+	−0.96	NA	NA	NA	NA	6	−211.45	435.26	99.16	0.0
PB2	1.50	NA	−0.96	NA	NA	NA	NA	4	−218.40	444.97	108.87	0.0
PB1	1.90	+	NA	NA	NA	NA	NA	5	−257.21	524.68	188.57	0.0
PB0	1.49	NA	NA	NA	NA	NA	NA	3	−264.15	534.41	198.30	0.0

Abbreviations: AIC_C_, second-order Akaike Information Criterion; δAIC_C_, delta AIC_C_; Int, intercept; LMM, linear mixed effects model; logLik, logarithmic likelihood; Mod, model; NA, not available; PBF, phytoplankton biomass model F; PB8, phytoplankton biomass model 8; PB7, phytoplankton biomass model 7; PB6, phytoplankton biomass model 6; PB5, phytoplankton biomass model 5; PB4, phytoplankton biomass model 4; PB3, phytoplankton biomass model 3; PB2, phytoplankton biomass model 2; PB1, phytoplankton biomass model 1; PB0, phytoplankton biomass model 0.

### Hypothesis 2

Consistent with our second hypothesis and the patterns observed for phytoplankton biomass (Fig 2A), there was a strong trophic cascade in the warm ecosystems by the end of the experiment (Fig 2B). The trophic cascade became apparent after the first weeks of the experiment and strengthened over time and with temperature (Fig 2B) (Table 2). The best model included a term for mean ecosystem temperature (T_M_) as well as week (T_w_), and a week × temperature interaction. By week 9, the trophic cascade increased exponentially with temperature (Fig 2B) to an estimated *E*_*TC*_ = 0.77 (estimated from the fixed effects model shown in Table 2, plus random effect).

**Table 2:** Model selection results for trophic cascade analysis. We used LMMs with terms for average temperature for ecosystem *j* in week *w* (T_wj_), weeks 2–9 (Wk), and their interaction. We treated the power level (e.g., 100 W, 200 W, etc.), our temperature treatment, as a random effect to account for repeated measures on ecosystems over time. We compared models using likelihood ratios (LogLik), AIC_C_, Akaike weights (w), and δAIC_C_ weights. The model was fit to 79 observations in 10 groups. The full model (TCFull) includes all terms, and models representing alternate hypotheses excluded terms indicated by “NA.” Coefficients were pooled (Methods: Statistical analysis) to estimate slopes and intercepts for Fig 2.

Mod	Int	T_wj_	Wk	T_wj_*Wk	df	logLik	AIC_C_	δAIC_C_	w
TCFull	0.19	−0.01	0.12	0.11	6	−60.78	134.73	0.00	0.77
TCmC	0.02	−0.74	0.14	NA	5	−63.26	137.33	2.60	0.21
TCmE	0.56	NA	0.04	NA	4	−67.36	143.27	8.54	0.01
TCmF	0.79	NA	NA	NA	3	−68.80	143.92	9.19	0.01
TCmD	0.79	−0.05	NA	NA	4	−68.76	146.07	11.34	0.00

Abbreviations: AIC_C_, second-order Akaike Information Criterion; δAIC_C_, delta AIC_C_; Int, intercept; LMM, linear mixed effects model; logLik, logarithmic likelihood; Mod, model; NA, not available; TCmC, Trophic Cascade Model C; TCmD, Trophic Cascade Model D; TCmE, Trophic Cascade Model E; TCmF, Trophic Cascade Model F.

**Fig 4:**
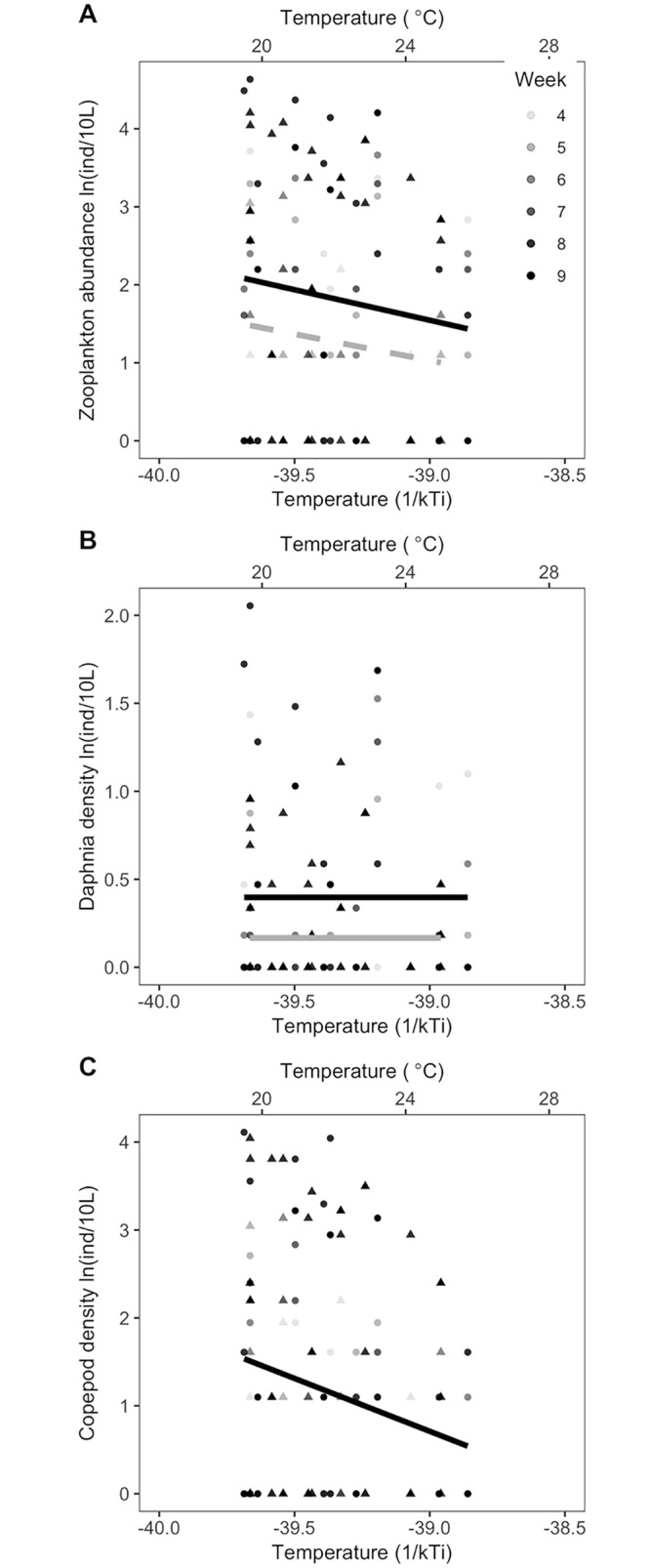
(**A**) Total zooplankton density (ln(ind)/10 L), comprising *Daphnia* and copepod taxa, declined with increasing temperature but not with predator presence. (B) *Daphnia* density (ind/L) declined with predators (gray dashed line, versus black line indicating trend with no predators) (Table 4), and (C) copepod spp. density (ln(ind)/10 L) declined with temperature but not predators (Table 5). Lines are regressions, with ecosystem as a random effect for ecosystems with predators (gray lines) and without predators (black solid line). Each data point is an observed total zooplankton density for crustacean taxa (*Daphnia* and copepods) in each ecosystem on a sampling date. Data for these figures may be found at https://doi.org/10.5281/zenodo.2652579 in GarzkeAllwks.csv. ind, individuals.

We find additional evidence of temperature-dependent trophic interactions in the responses of zooplankton grazer assemblages to warming and predation. Total zooplankton density declined with increasing temperature (Table 3; *E*_*ZP*_ = 0.73 95% CI: −0.93–2.21, based on model conditional averaged estimates from linear regression of ln-transformed densities; Fig 4). Predators reduced ln(density) of *Daphnia* (*Z*_*j*_ = −0.23 ± 0.06), the dominant grazer, and there was no apparent effect of temperature on *Daphnia* density (Table 4). Copepod density declined with temperature (*E*_*C*_ = 1.21 95% CI: −1.07 to −1.41) and not in response to predation (the best model did not include a predation term, Table 5). We measured the zooplankton standard length for 641 individuals of all ages. Mean length was 0.72 cm, and sizes ranged from 0.34 to 1.94 cm. We did not observe a decline in body size with temperature (the best model did not include a temperature term, S2 Table), as would be expected by a hypothesis based on the temperature size rule. Predation reduced total zooplankton body size, driven by size shifts in *Daphnia* (S2 Table).

**Table 3:** Zooplankton density. Results of model selection for zooplankton abundance in ecosystems with grazers (AG) and with grazers and predators (AGP). We used linear regressions (Methods: Statistical analysis). Models included terms for weekly average temperature (T_wj_), ecosystem trophic treatment (Z_j_) and their interaction, and a random effect for ecosystem identity. We modeled 120 observations in 20 groups (ecosystems). We compared models using likelihood ratios (LogLik), AIC_C_, Akaike weights (w), and δAIC_C_ weights. NA indicates that the term was not included in the model.

Mod	Int	T_wj_	Z_j_	T_wj_* Z_j_	df	logLik	AIC_C_	δAIC_C_	w
Z1c	1.56	NA	NA	NA	3	−218.17	442.56	0.00	0.22
Z1	1.82	0.78	+	+	6	−214.92	442.58	0.02	0.22
Z1b	1.56	0.66	NA	NA	4	−217.17	442.69	0.14	0.20
Z1d	1.82	0.74	+	NA	5	−216.18	442.89	0.34	0.18
Z1a	1.81	NA	+	NA	4	−217.30	442.96	0.40	0.28

Abbreviations: AG, algae and grazers; AGP, algae, grazers, and predators; AIC_C_, second-order Akaike Information Criterion; δAIC_C_, delta AIC_C_; Int, intercept; logLik, logarithmic likelihood; Mod, model; NA, not available; Z1, zooplankton abundance model 1; Z1a, zooplankton abundance model a; Z1b, zooplankton abundance model b; Z1c, zooplankton abundance model c; Z1d, zooplankton abundance model d.

**Table 4:** *Daphnia* density: Results of model selection for *Daphnia* abundance in ecosystems with grazers and with grazers and predators. We used linear regressions (Methods: Statistical analysis). Models included terms for weekly average temperature (T_wj_), ecosystem trophic treatment (Z_j_) and their interaction, and a random effect for ecosystem identity. We compared models using likelihood ratios (LogLik), AIC_C_, Akaike weights (w), and δAIC_C_ weights. We modeled 120 observations in 20 groups (10 AGP ecosystems with predators, and 10 AG ecosystems without predators). NA indicates that the term was not included in the model.

Mod	Int	T_wj_	Z_j_	T_wj_* Z_j_	df	logLik	AIC_C_	δAIC_C_	w
D1a	0.40	NA	+	NA	4	−75.77	159.9	0.00	0.41
D1c	0.28	NA	NA	NA	3	−76.88	160.0	0.07	0.39
D1d	0.40	0.15	+	NA	5	−76.20	162.9	3.02	0.09
D1b	0.28	0.11	NA	NA	4	−77.35	163.0	3.15	0.08
D1	0.40	0.20	+	+	6	−76.12	165.0	5.10	0.03

Abbreviations: AG, algae and grazers; AGP, algae, grazers, and predators; AIC_C_, second-order Akaike Information Criterion; δAIC_C_, delta AIC_C_; D1, *Daphnia* abundance model; D1a, *Daphnia* abundance model a; D1b, *Daphnia* abundance model b, D1c, *Daphnia* abundance model c; D1d, *Daphnia* abundance model d; Int, intercept; logLik, logarithmic likelihood; Mod, model; NA, not available.

**Table 5:** Copepod density: Results of model selection for copepod spp. abundance in ecosystems with grazers and with grazers and predators. We used linear regressions (Methods: Statistical analysis). Models included terms for weekly average temperature (T_wj_), ecosystem trophic treatment (Z_j_) and their interaction, and a random effect for ecosystem identity. We compared models using likelihood ratios (LogLik), AIC_C_, Akaike weights (w), and δAIC_C_ weights. We modeled 120 observations in 20 groups (10 AGP ecosystems with predators, and 10 AG ecosystems without predators). NA indicates that the term was not included in the model.

Mod	Int	T_wj_	Z_j_	T_wj_* Z_j_	df	logLik	AIC_C_	δAIC_C_	w
C1b	1.159	1.20	NA	NA	4	−199.71	407.8	0.00	0.55
C1	1.189	1.32	+	+	6	−198.88	410.5	2.74	0.14
C1d	1.187	1.21	+	NA	5	−200.01	410.5	2.77	0.14
C1c	1.159	NA	NA	NA	3	−202.17	410.5	2.78	0.14
C1a	1.163	NA	+	NA	4	−202.40	413.2	5.39	0.04

Abbreviations: AG, algae and grazers; AGP, algae, grazers, and predators; AIC_C_, second-order Akaike Information Criterion; δAIC_C_, delta AIC_C_; C1, copepod abundance model; C1a, copepod abundance model a; C1b, copepod abundance model b; C1c, copepod abundance model c; C1d, copepod abundance model d; Int, intercept; logLik, logarithmic likelihood; Mod, model; NA, not available.

### Hypothesis 3

**Fig 5:**
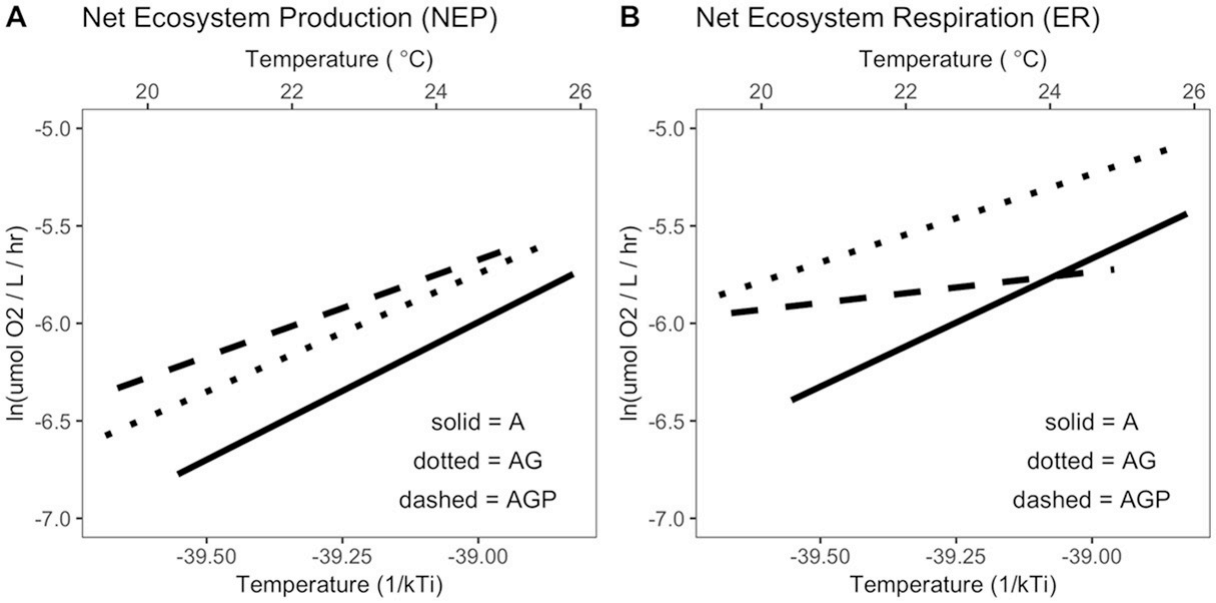
The effect of mean ecosystem temperature on (A) NEP and (B) net ER for three community types that varied in their trophic interactions: (i) algae-only (A), (ii) algae + grazers (AG), and (iii) algae + grazers + notonectid predators (AGP). Black lines indicate the among-ecosystem effects of temperature, modeled by Eq 5 using hierarchical regressions fit to among-ecosystem variation in temperature, after taking into account within-group variation temperature effects (light lines) (Tables 1, 6 and 7). Temperature dependences within and among tanks were estimated by best model or best model set (Tables 1, 6 and 7, Methods: Statistical analyses). Temperature in Celsius is shown for comparison only; models were fit to inverse temperature. All measured data points to which models were fitted are shown in Fig 6. Temperatures within tanks declined over time (S2 Fig). Data for these figures may be found at https://doi.org/10.5281/zenodo.2652579 in GarzkeAllwks.csv. A, algae only; AG, algae and grazers; AGP, algae, grazers, and predators; ER, ecosystem respiration; NEP, net ecosystem production.

**Fig 6:**
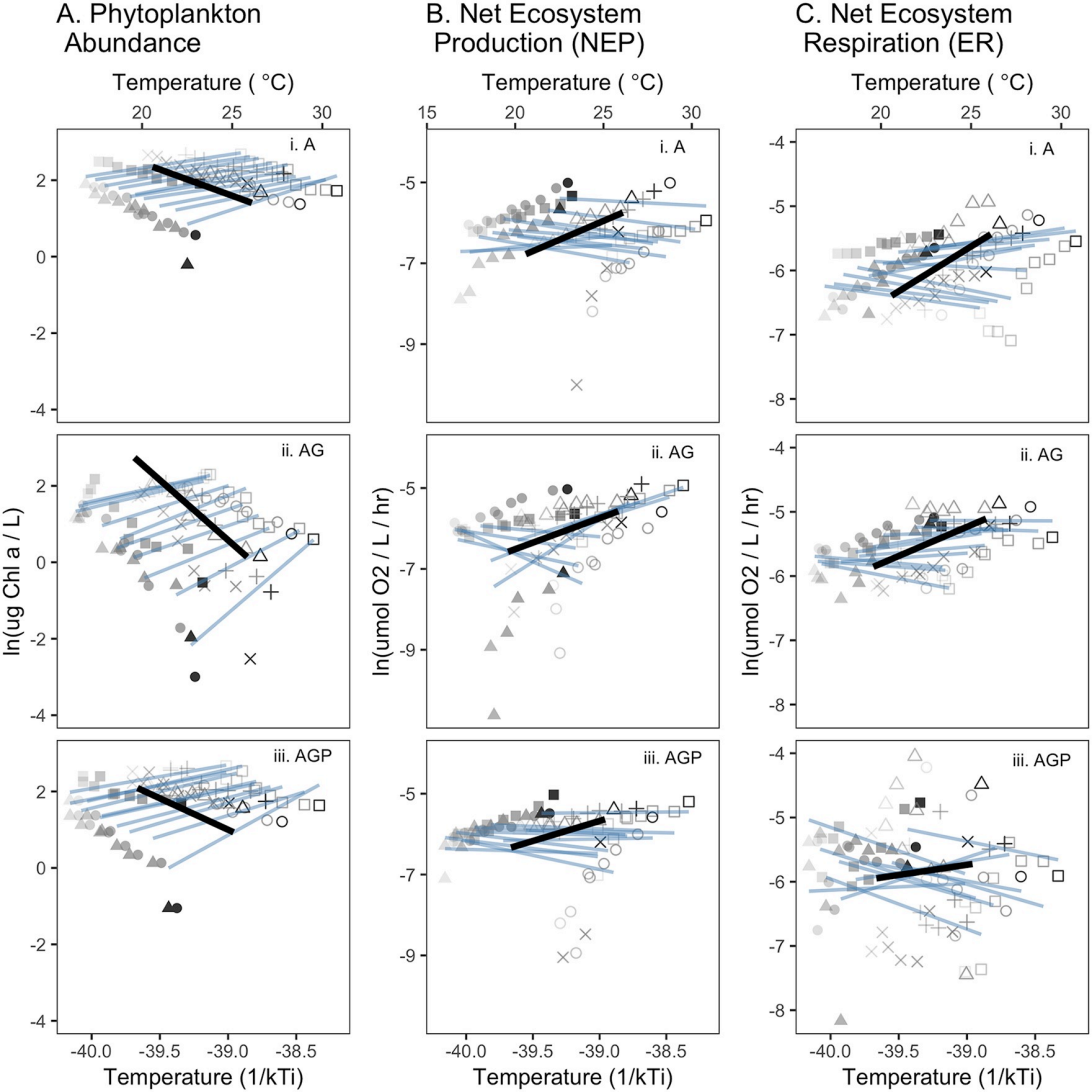
The effect of ecosystem temperature (T_wj_) on (A) phytoplankton biomass, (B) NEP, and (C) net ER for three community types that varied in their trophic interactions: (i) algae-only (A), (ii) algae + grazers (AG), and (iii) algae + grazers + notonectid predators (AGP). There were 10 ecosystems (*j*) in each trophic treatment, and each ecosystem was sampled 8 times (once per week from weeks 2 to 9). Each week is indicated by a symbol shape, and ecosystem identities within weeks are distinguished by shades of gray. In a single model (Eq 13), we considered effects of temperature within ecosystems over time, as well as among-ecosystem variation in mean temperature (Figs 2 and 5). Blue lines are fit to the 8 observations (points) from each ecosystem (one from each week), and their slope indicates within-ecosystem temperature effects estimated from best models in Tables 2, 6 and 7. Black lines indicate the modeled among-ecosystem effects of temperature (Tables 1, 6 and 7; Figs 2A and 5). Temperature in Celsius is shown for comparison only; models were fit to inverse temperature. Temperatures within tanks declined over time (S2 Fig). Data for these figures may be found at https://doi.org/10.5281/zenodo.2652579 in GarzkeAllwks.csv. A, algae only; AG, algae and grazers; AGP, algae, grazers, and predators; ER, ecosystem respiration; NEP, net ecosystem production.

Across ecosystems, higher temperatures increased NEP and ER (Tables 6 and 7; Fig 5). The LMM for NEP (Table 6) suggests that ecosystem temperature and trophic structure interact to influence ecosystem oxygen fluxes, yet their estimated temperature dependences did not appear to differ when confidence intervals were compared (Fig 3). The estimated across-system temperature dependence of NEP was the strongest in algae-only communities (Fig 5), and confidence intervals for the temperature dependence term include 0 for the systems with predators (Fig 3). ER increased with temperature across ecosystems (Fig 5), and this effect did depend on trophic structure (Table 7). The estimated temperature dependence on NEP and ER was strongest in the algae-only systems and weakest in systems with predators (Fig 3). See Fig 6 for all data.

**Table 6:** Results of model comparisons for effects of temperature and time on NEP based on AIC weight (*w*) and δAIC_C_ values. Nested versions of the full model (Methods, Eq 12). Response variables are modeled as functions of temperature T_wj_ for each tank *j* on week *w* relative to the mean temperature $T_{M}$ for tank *j* over all weeks (T in Kelvin), and trophic structure (*Z*_*j*_). Models included a random effect for the experimental unit—tanks with and without predators received the same power inputs. See Methods for additional details on modeling. The model was fit to 219 observations in 30 groups. The full model (NEPF) includes all terms, and models representing alternate hypotheses excluded terms indicated by “NA.” Values indicate model-estimated coefficients. Coefficients were pooled (Methods: Statistical analysis) to estimate slopes and intercepts for Figs 3 and 5.

Mod	Int	Z_j_	T_wj_	T_M_	T_wj_*Z_j_	T_M_*Z_j_	T_wj_*T_M_	df	logLik	AIC_C_	δAIC_C_	w
NEP8	−6.42	+	0.29	−1.40	NA	+	+	11	−266.47	556.21	0.00	0.39
NEPF	−6.42	+	0.37	−1.42	0.84	+	+	12	−265.54	556.60	0.39	0.32
NEP7	−6.41	+	0.03	−1.39	NA	NA	+	9	−269.68	558.22	2.01	0.14
NEP3	−6.15	NA	0.02	−0.96	NA	NA	NA	5	−274.36	559.01	2.80	0.10
NEP4	−6.15	NA	0.02	−0.96	0.61	NA	NA	6	−273.86	560.12	3.91	0.05
NEP0	−6.15	NA	NA	NA	NA	NA	NA	3	−283.15	572.41	16.20	0.00
NEP2	−6.15	NA	0.03	NA	NA	NA	NA	4	−283.13	574.44	18.23	0.00
NEP1	−6.26	+	NA	NA	NA	NA	NA	5	−282.25	574.78	18.57	0.00
NEP6	−6.26	+	0.27	NA	NA	+	NA	8	−279.83	576.34	20.13	0.00
NEP5	−6.26	+	0.03	NA	NA	NA	NA	6	−282.23	576.85	20.64	0.00

Abbreviations: AIC_C_, second-order Akaike Information Criterion; δAIC_C_, delta AIC_C_; NEPF, NEP full model; NEP0, NEP model 0; NE01, NE0 model 1; NEP2, NEP model 2; NEP3, NEP model 3; NEP4, NEP model 4; NEP5, NEP model 5; NEP6, NEP model 6; NEP7, NEP model 7; NEP8, NEP model 8; Int, intercept; logLik, logarithmic likelihood; Mod, model; NA, not available. NEP, net ecosystem production.

**Table 7:** Results of model comparison for effects of temperature and time on ER based on AIC weight (w) and δAIC_C_ values. Nested versions of the full model (Methods, Eq 12). Response variables are modeled as functions of temperature T_wj_ for each tank *j* on week *w* relative to the mean temperature *T*_*M*_ for tank *j* over all weeks (T in Kelvin), and trophic structure (Z_j_). Models included a random effect for the experimental unit—tanks with and without predators received the same power inputs. The model was fit to 240 observations in 30 groups. See “Methods: Statistical analyses” for additional details on modeling.

Mod	Int	Z_j_	T_wj_	T_M_	T_wj_*Z_j_	T_M_*Z_j_	T_wj_*T_M_	df	logLik	AIC_C_	δAIC_C_	w
ER7	−6.09	+	0.11	−1.32	NA	+	NA	9	−185.88	390.54	0.00	0.60
ER8	−6.09	+	0.02	−1.32	+	+	NA	11	−184.58	392.31	1.77	0.25
ERF	−6.09	+	0.06	−1.32	+	+	0.42	12	−183.97	393.31	2.77	0.15
ER3	−5.79	NA	0.11	−0.67	NA	NA	NA	5	−201.37	413.00	22.46	0.00
ER4	−5.79	NA	0.11	−0.68	NA	NA	0.50	6	−200.45	413.27	22.73	0.00
ER1	−5.94	+	NA	NA	NA	NA	NA	5	−202.46	415.18	24.64	0.00
ER5	−5.94	+	0.11	NA	NA	NA	NA	6	−201.75	415.85	25.31	0.00
ER6	−5.94	+	0.02	NA	+	NA	NA	8	−200.49	417.60	27.06	0.00
ER0	−5.79	NA	NA	NA	NA	NA	NA	3	−207.04	420.17	29.63	0.00
ER2	−5.79	NA	0.11	NA	NA	NA	NA	4	−206.32	420.82	30.28	0.00

Abbreviations: AIC, second-order Akaike Information Criterion; δAIC_C_, delta second-order Akaike Information Criterion; ER, ecosystem respiration; ERF, ER full model; ER0, ER model 0; ER1, ER model 1; ER2, ER model 2; ER3, ER model 3; ER4, ER model 4; ER5, ER model 5; ER6, ER model 6; ER7, ER model 7; ER8, ER model 8; Int, intercept; logLik, logarithmic likelihood; Mod, model; NA, not available.

In addition to the variation among ecosystems in temperature that was the main focus of our hypotheses, the biotic and abiotic conditions in experimental ecosystems varied over time. Temperature varied within experimental ecosystems over time (S2 Fig). Overall, temperatures declined between the beginning and the end of the experiment, with some variation among weeks reflecting weather conditions. Phytoplankton community composition shifted over time (S1 Fig), but visual inspection of the species at each time point indicated no specific taxa driving the changes, and there was no association between phytoplankton species composition and temperature (S3 Fig). Chlorophyll *a* concentration declined over time in all treatments (S4 Fig). An LMM indicated that this decline was weakest in the algae-only treatments (S1 Table). Visual inspection of trends (S4 Fig) suggests that this decline over time was driven by the decline in all tanks in weeks 8 and 9, following a major rain event and drop in all tank temperatures (S2 Fig). When we reanalyzed the temporal trend for just weeks 2−7, excluding weeks 8−9, the trend in chlorophyll *a* over time approached 0 in A and AGP treatments, but still persisted in AG treatments (S1 Table).

Effects of temporal temperature variation on phytoplankton biomass within ecosystems differed starkly from effects of temperature among ecosystems (Fig 6). Within ecosystems, higher temperatures were associated with higher phytoplankton standing stocks (Fig 6A), opposite to the trend with temperature among ecosystems. NEP and ER varied with temperature within ecosystems, and there is some evidence that this temperature effect interacted with both the trophic structure treatments (Table 6, model NEP8; Table 7, model ER8).

## Discussion

Temperature affects the metabolic rates of all organisms, and per capita responses to temperature of many co-occurring individuals add up to nothing less than the biological component of ecosystem-scale carbon and oxygen flux. Understanding biological responses to temperature change across scales of organization (cells to the biosphere) is a major challenge in ecological research. Meeting this challenge requires joining theoretical frameworks and synthesizing empirical evidence of temperature effects across scales and systems. Despite much progress, there remains a gap between patterns that emerge in community-level experiments and the multiscale theoretical framework (MTE) that links temperature-dependent metabolism to larger-scale patterns for temperature dependence. Here, we aimed to test the hypothesis that the effects of temperature on ecosystem processes that reflect metabolic temperature dependence are not highly sensitive to local differences in the trophic structure of a community (e.g., presence or absence of a predator). This question draws upon ideas supported by the MTE and community ecology theory predicting that species interactions modify the effects of temperature on community structure and function. We found that in aquatic ecosystems characterized by the presence or absence of predator–prey species interactions, temperature-dependent trophic cascades only modestly altered the effects of temperature on net ecosystem oxygen production and consumption (NEP and ER). We found that higher average temperatures increased NEP and ER while total phytoplankton biomass declined, and all ecosystem-level temperature responses were stronger than expected for per capita temperature-dependent oxygen production or consumption.

Our first hypothesis was based on the expectation that our experimental systems would include top-down predator effects that altered phytoplankton standing stock, and possibly interacted with temperature to influence algal size distributions or other traits. We found that trophic structure did modify the effect of temperature on phytoplankton biomass, failing to reject our first hypothesis. The decline in phytoplankton standing stocks that we observed with warming across ecosystems is consistent with theoretical expectations that in closed systems with limited resources, increases in per capita metabolic rates with temperature could lead to declines in standing stocks [15,18,39,46]. Phytoplankton standing stocks responded most strongly to temperature in the communities with grazers but no predators, suggesting that temperature-dependent grazing can exacerbate the temperature dependence of algal standing stocks. Overall, the temperature dependence of phytoplankton standing stocks greatly exceeded expectations based on temperature dependence of per capita photosynthesis or respiration rates (Fig 3). Our hypothesis (Eq 3) allowed for changes in phytoplankton standing stocks to be explained by direct effects of temperature on per capita metabolism, as well as effects of temperature on thermal traits, density, or body size distributions. We suggest that change in per capita metabolic response and density were the primary components of this change. We did not observe clear shifts in the species composition of the phytoplankton assemblage with temperature; still, we do not have high-resolution data on phytoplankton cell size or traits, so we cannot reject these mechanisms as contributors to the patterns we observe.

Our second hypothesis, based on recent experimental results in other freshwater and grassland systems, was that the trophic cascade would get stronger as ecosystem temperatures warmed. We found support for this hypothesis in our system, providing the first evidence that trophic cascade strength increases continuously with temperature. Prior to our study, evidence of stronger trophic cascades with warming were from experiments that test two temperature levels, an ambient and a simulated future scenario of approximately +3 °C [29,47,48]. We show here that this pattern continues over a thermal range of 10 °C. The indirect effects of predators on phytoplankton biomass appear to have been mediated by predation on the dominant grazer, *Daphnia*. Predators reduced *Daphnia* density and thereby shifted grazer assemblages toward the less effective copepod grazers at all temperatures. This trophic cascade, mediated by shifts in grazer composition as well as total density, is a classic food web motif in freshwater systems [43]. Interestingly, at warmer temperatures grazer density was lowest, yet we still observed declines in biomass of phytoplankton. This pattern could reflect higher per capita grazing by the remaining grazer individuals. Algal productivity rates are an important element of trophic cascade strength [15,34], and higher NEP at warmer temperatures would contribute to a stronger trophic cascade, even as grazer density declines. As with hypothesis 1, we infer that the effect of temperature on the trophic cascade strength reflects not only the effect of temperature on per capita metabolic rate but also shifts in algal traits or body sizes, or both.

We tested a third hypothesis, that the effects of temperature on biomass and trophic cascade strength would lead to distinct relationships between temperature and NEP and ER for each trophic treatment type (e.g., with versus without predators). We found that the effect of temperature on phytoplankton standing stock was much greater than the effects of temperature on NEP or ER. For NEP and ER, there was support for a model with an interaction between trophic structure and mean temperature, but for NEP a model without the interaction was ranked highly (Table 6), and confidence intervals for the pooled estimated temperature dependence do not indicate differences in temperature dependences among trophic treatments. Therefore, the strong effects of temperature on community structure (biomass, trophic cascade strength) did not translate directly to temperature effects on net ecosystem flux rates.

The estimated temperature dependences of NEP and ER were greater than expected based on temperature-dependent per capita, mass-normalized respiration, and photosynthesis metabolic rates. It is well established that temperature dependence of aerobic respiration is approximately *E*_*R*_ = 0.65 eV, and that this value explains the temperature dependence of mass-normalized ecosystem metabolism at the ecosystem scale [2–4]. The temperature dependence of photosynthesis at suboptimal temperatures appears to be *E*_*PS*_ = 0.32 eV for algal systems (although *E*_*PS*_ values of 0.65 eV are also observed), and this can emerge at population [39] and ecosystem scales [4] in aquatic systems, suggesting *E*_*NEP*_ = 0.32–0.65 eV [10,20,49]. Across our experimental temperature gradient, we observed values of *E*_*R*_ > 0.65 eV for both NEP and ER, although confidence intervals for ER did include this value (Fig 3) for algae-only ecosystems. These results led us to reject the “first-order metabolic theory” hypotheses that temperature dependence of ecosystem functions scales directly with general temperature dependence of metabolism. Our results further suggest that changes in species interactions within communities, such as loss or gain of a predator species, could alter the responses of net ecosystem fluxes to temperature changes.

Temperature had a stronger effect on phytoplankton standing stock than on NEP. This difference in phytoplankton biomass and oxygen-flux responses to temperature could reflect several processes operating at different scales of organization. First, we expect that per capita rates of oxygen flux increase with warming, so that a given biomass of phytoplankton can be more productive at warmer temperatures if resources are not limiting [4,46,50]. Patterns at the ecosystem scale could deviate from expectations based on direct metabolic scaling of per capita metabolism if size distributions shift toward smaller cells, as is common with warming, as described by the temperature size rule [23,51]. The allometric scaling of metabolic rate with body size (Eq 2) predicts greater oxygen flux for a given total biomass comprised of small individuals. The distribution of thermal tolerance phenotypes may have shifted within the phytoplankton communities. Three months may be sufficient time for evolutionary change [52]. We did not see clear evidence of shifts in species composition with temperature, and it is likely that the species we collected to inoculate our ecosystems were able to tolerate our experimental conditions because we collected them from a shallow lake in Vancouver in which the water temperature likely tracks summertime air temperatures, therefore experiencing temperatures between 19 and 30 °C. Our experimental ecosystems likely did not expose zooplankton to temperatures outside what they would have experienced in a natural system, and we therefore assume they were adapted to these conditions.

In addition to the effects of temperature on per capita metabolism and size structure, at the ecosystem scale, effective resource supply may have changed with temperature, violating an implicit assumption of Eqs 1–4. Even though these were closed ecosystems with regard to external influxes of nutrients, and they experienced the same light conditions, internal nutrient processes could have varied with temperature in ways that made nutrients more available in warmer ecosystems. For example, our ecosystems did not include a benthic habitat that can store nutrients and organic material and slow down nutrient cycling. Heterotrophic microbial processes responsible for rapid nutrient turnover would be accelerated by temperature, perhaps making nutrients available in warmer systems more than in colder systems. Another potential, and speculative, explanation for higher productivity than expected in warmer ecosystems is that some algae species are capable of biological nitrogen fixation [53], and this activity is more feasible at higher temperatures. These two biological processes that are themselves temperature dependent could create a resource gradient in parallel with the temperature gradient [15,50], leading to higher than expected NEP at warmer temperatures relative to the same ecosystem at cooler temperatures.

Although there was no benthic sediment in our ecosystems, algae likely colonized the sides and bottom of the tanks. Benthic algae may also have contributed to NEP and ER estimates in our systems [54]. We did not observe notable amounts of accumulated benthic algae, but even small amounts could have contributed to total ecosystem fluxes and led to covariation in total biomass with temperature. If the ratio of phytoplankton to benthic algae was temperature-dependent [54], our primary producer biomass estimates may have increasingly underrepresented total algal biomass at higher temperatures. To be conservative, we did not present mass-normalized NEP estimates because we could not normalize to any benthic algal metabolic biomass. Covariation between biomass and temperature is common across geographic variation in temperature [12,20,53] and therefore present in other estimates of NEP across broad spatial scales when biomass cannot be estimated well.

Across mean ecosystem temperatures of 19–30 °C, we observed no sign of ecosystem collapse or threshold responses to warming. Changes in community structure and the increase in trophic control along the temperature gradient appear to be exponential and monotonic over the 10 °C gradient (Eq 2), suggesting that linear (or additive) models of temperature effects in most warming experiments, which only test two or three temperatures, may underestimate warming effects over broader thermal gradients. We observed little evidence of abrupt transitions that might be expected if thermal stress responses by individual phenotypes drove ecosystem-scale responses. We did observe declines in grazer density with warming even in the absence of predators, suggesting there were direct or indirect negative effects of temperature on grazers. But we did not see clear shifts in algal species composition among treatments, suggesting that no species group was exposed to temperatures above its critical thermal maximum. Another challenging aspect of warming experiments at the population and community scales is interpreting patterns in the context of transient dynamics. Our ecosystems certainly did not reach long-term states, because varying weather conditions and multi-week generation times of zooplankton would have precluded that. Still, we did not observe signs of transient dynamics in these communities over time, such as population cycles or abrupt changes.

In our systems, algal biomass and zooplankton abundance in food webs were more resistant to temperature in the presence of longer food chains. Predators reduced zooplankton density and caused a clear trophic cascade. Trophic control, and therefore any mitigating effects of predators on biomass change, was weak at low temperatures and increasingly strong at higher temperatures (A versus AG treatment, Fig 3). This pattern is consistent with previous findings that ecosystem functions in systems with two (or even numbers of) trophic levels tend to be more sensitive to warming than systems with odd numbers, due to cascading effects of predation on primary producers [48]. Additionally, in our experiment, predators were not dynamically responsive; they did not have time to reproduce during the experiment. Consequently, they represent mortality for zooplankton that may have varied with temperature effects on per capita predation rates by predators, but not a dynamic demographic response that could lead to different outcomes for prey [55]. In many systems, predators are subsidized by other habitats and food sources, and their populations are not dynamically coupled to prey [56]. In fact, this decoupling has been shown to be important in thermally stratified lakes [57]. Inferences drawn based on this experiment about how species interactions affect community and ecosystem responses are restricted to systems with dynamics in the primary producers and primary consumers, with predation-related mortality imposed by a third trophic level through per capita consumption effects but not population dynamics of the predators.

The growing literature of experimental tests of how warming affects interacting species aims to reduce uncertainty in projected ecological changes associated with climate change. Warming experiments have shown a wide variety of consequences for species interactions, from shifts in community composition, strengthening top-down control, and shifts in body size [16,18,54]. We have shown that these shifts do alter the effects on the temperature dependence of net ecosystem oxygen production and consumption as modeled by the MTE, but that these models may be extended to consider community-level changes. By measuring community and ecosystem responses over a broad thermal gradient under controlled conditions, we have provided empirical evidence that large effects of temperature on community biomass can occur in the context of less strong effects of temperature on net ecosystem function. This is a step toward closing the gap between patterns observed across ecosystems that appear to reflect effects of temperature on metabolic rates, and observations at intermediate scales that temperature can have large effects on the abundance of species. Taken together, these results suggest our efforts to predict community change with warming may benefit from the general metabolic scaling theory framework to understand even local-scale effects of temperature change at the community level.

## Methods

### Experimental design and setup

We assembled freshwater food webs in 30 outdoor mesocosms (370-L tanks) at the University of British Columbia, Vancouver, Canada (49°14’52” N, 132°13’57” W). Mesocosms were filled with municipal water on June 26, 2012, heaters were added, and filled tanks were left for 1 week to allow chlorine to evaporate before organisms were introduced. We experimentally manipulated temperature (10 levels) and trophic structures (algae-only [A], algae + grazer [AG], and algae + grazer + predator [AGP], Fig 1). There was one tank per temperature per trophic treatment; statistical power was derived from the regression design rather than replication within treatment levels (see Methods: Statistical analysis). We monitored temperature continuously and sampled biotic variables once per week for 9 weeks. Tanks were arranged randomly in space with regard to treatment. The spatially randomized assignment of temperature and trophic treatments eliminated systematic variation in negligible allochthonous carbon inputs.

On July 2, 2012, mesocosms were inoculated with pond water (1 L) from the UBC Pond Facility containing living algae, collected and filtered through a 64-μm sieve to remove zooplankton and larvae. Three days later, we collected zooplankton at Trout Lake, Vancouver, BC (49°15’23” N, 123°03’44” W), with a vertical tow net (64-μm mesh). Zooplankton were mixed in buckets to homogenize species composition, were introduced to mesocosm temperatures over a 12-hour gradual acclimation period to avoid stress associated with an abrupt temperature change, and dead organisms were removed. Initial experimental communities consisted of 25 phytoplankton taxa (S2 Table), and those with zooplankton included predominantly two zooplankton taxa (cladocerans *Daphnia* sp. and calanoid copepod *Eurytemora* sp.) and, rarely, cyclopoid copepods. To ensure colonization of grazing zooplankton, in addition to the random aliquot of zooplankton added to each zooplankton ecosystem (all algae-grazer and algae-grazer-predator ecosystems), we added two individuals of *Daphnia* sp. and ten *Eurytemora* sp. Thus, each zooplankton community began with at least 12 grazing zooplankton individuals. We introduced two individual notonectid predators (*Notonecta undulata*), collected from ponds at the UBC Pond Facility, on July 4, 2012 to 10 algae-grazer-predator tanks. Notonectids generate trophic cascades by suppressing zooplankton [58]. Notonectids did not reproduce during the experiment, and we replaced dead notonectids during the experiment with similar-sized individuals from the same source population.

We added 160 μg NaNO_3_ L^−1^ and 10 μg KH_2_PO_4_ L^−1^ to each tank (16:1 N:P) on July 3, 2012. These quantities of nutrients represent typical deposition inputs to similar lakes [59]. Water was heated with submersible aquarium heaters (50-, 100-, 150-, 200-, 250-, 300-, 350-, 400-, and 450-watt) to increase temperature above ambient daily temperature. Temperatures were recorded hourly using Thermochron iButton data loggers. Data loggers were suspended in the middle of the tanks, approximately halfway between the surface and the bottom. Temperature differences among tanks were consistent throughout the course of the experiment (S2 Fig). Heaters were placed at the bottom of the mesocosms. Mesocosms were covered with two layers of window screen to minimize colonization by other invertebrates. Water levels were maintained by natural precipitation and weekly additions to maintain volume.

### Plankton sampling and analysis

We sampled phytoplankton, chlorophyll *a*, zooplankton, and oxygen concentrations weekly until August 28, 2012. We sampled algal assemblages in 100-mL water samples collected from approximately 40 cm below the surface. We counted and identified cells using the Utermöhl sedimentation method [60] and identified algae species or taxon level by inverted microscopy. We estimated chlorophyll *a* concentration using a Trilogy fluorometer (Turner Designs). Chlorophyll *a* concentration can be used as a proxy for biomass, and although the ratio between chlorophyll *a* and total biomass can itself vary with temperature, size, and species composition [61,62], the chlorophyll *a* concentration represents biomass allocated to photosynthesis and NEP, our measure of ecosystem function. We measured oxygen concentrations in situ using YSI-85 oxygen sensor (Yellow Springs Instruments, Yellow Springs, OH).

We collected zooplankton samples using a “depth integrated zooplankton sampler.” The device is a cylinder 4 cm in diameter and 60 cm in length with a cap at one end. We mixed mesocosm water gently, then submerged the sampler vertically, sealed it, removed it, and dumped water into a bucket. We repeated until we had removed 10 L of water, which was then filtered through a 64-μm mesh to collect zooplankton, and then the filtered water was returned to mesocosms. Plankton was fixed with Lugol’s iodine solution (5%). Under 10× magnification, we counted and identified zooplankton to genus level and measured the standard length for all development stages in weeks 4−8.

### Estimation of biomass and oxygen fluxes

We estimated whole ecosystem oxygen fluxes using the dissolved oxygen (DO) change technique [63]. Oxygen production during the daytime is the product of photosynthesis minus respiration (NEP), and oxygen depletion during the night is the result of respiration (ER). We compared DO concentrations measured over 24 hours (dawn, dusk, and the following dawn). Comparison of oxygen concentrations at dawn, dusk, and dawn of the following day (Eq 5) can indicate not only the cumulative biotic NEP and ER fluxes during that time interval but also differences in water temperature that affect oxygen concentrations in water. At standard pressure, which is appropriate for our experiment near sea level, oxygen saturation can change by approximately 1 mg/L with a change in temperature of 5 °C, described by

$${{[O}_{2}]}_{E}=e^{\left({\left[O_{2}\right]}_{water}-{\left[O_{2}\right]}_{sat}*\text{ln}\left(T+45.93\right)\right)},$$ (5)

where [O_2_]_water_ is the O_2_ concentration of water, [O_2_]_sat_ is the concentration the water would have if it were at equilibrium with the atmosphere (390 μatm), and T is temperature of the observation (°C) [64]. For the differences in temperature we observed, corrections were on the order of mean 0.0002 ± SD 0.0008 μmol O_2_/L/hour for NPP, and mean 0.0008 ± SD 0.0003 μmol O_2_/L/hour for ER. Because these values are within 25% of our total observed changes in oxygen during those periods (mean 0.003 ± SD 0.001 μmol O_2_/L/hour for NEP and mean 0.003 ± SD 0.002 μmol O_2_/L/hour for ER), we included the correction in our analyses. Overall, the conclusions based on model selection did not depend strongly on the use of the correction.

We estimated NEP and ER by converting changes in observed O_2_ (mg L^−1^) between daytime observation times (*t*_*dawn*_, *t*_*dusk*_) and overnight observations (*t*_*dusk*_, *t*_*dawn2*_) to micromolar concentration (*z* = 31.25 μmol/1 mg), and correcting for changes in estimated equilibrium oxygen concentration ([*O*_2_]_*E*_) (Eq 5) due to changes in saturation state with temperature at each time:

$$NEP=[\frac{\left({{[O}_{2}]}_{dusk}{{-[O}_{2}]}_{dawn})-({{[O}_{2}]}_{Edusk}-{{[O}_{2}]}_{Edawn}\right)}{z*(t_{dusk}-t_{dawn1}}]$$ (6)

$$ER=[\frac{\left({{[O}_{2}]}_{dawn2}{{-[O}_{2}]}_{dusk})-({{[O}_{2}]}_{Edawn2}{{-[O}_{2}]}_{Edusk}\right)}{z*(t_{dawn2}-t_{dusk})}].$$ (7)

### Model and hypothesis development

The expression of temperature effects on a per capita metabolic rate *b*_*i*_—in our case, oxygen production via photosynthesis or consumption via respiration—in this model is a special case of a more complex equation that allows each species to follow a thermal performance curve (TPC), often described by a modified Sharpe-Schoolfield equation [10,20,65], in which an individual’s or population’s performance declines at high temperatures above some optimal temperature. We do not use this TPC model here for two reasons: we do not expect photosynthesis or respiration to exceed optimal operating temperatures in our system for most taxa, based on the fact that we collected them locally from a lake and habitat type (shallow pond) near the experimental site. We model our system using equations based on Eq 2. We believe this simpler exponential model is a suitable hypothesis for cross-system comparison in which community phenotypes or taxonomic composition may turn over along the thermal gradient [17,23]. We do not have thermal performance data for the many species in our communities that would allow fitting of TPCs within communities to test an alternate approach.

We modeled M_B_ (Eq 3) by including a term for trophic treatment (*Z*_*j*_) in the intercept term (Eq 3 rearranged and log transformed):

ln(MB)=ln(BRZj*b0(TC)〈mBα-1〉)+EMB(1kTj-1kTC) (8)

We derived the expression for the trophic cascade by relating algal biomass in the AGP and AG treatments:

MB.AGPMB.AG=BR.AGPeER(1kT-1kTC)(b0(TC))AGP〈mBα-1〉AGPBR.AGeER(1kT-1kTC)(b0(TC))AG〈mBα-1〉AG (9)

We then simplified and added temperature dependence of mass (*E*_*m*_) and normalization constants (*E*_*b*_). In the absence of additional information about their functional forms, we used general Arrhenius functions, but we note that other functions could be used if appropriate. Consequently, the ratio of *M*_*B*_ with and without predators may vary with temperature according to the relative temperature dependences of thermal traits and size distributions:

$$\frac{M_{B.AGP}}{M_{B.AG}}\propto \frac{{b_{0}(T_{C})}_{AG}e^{-E_{b.ag}/kT}{\langle m_{B}^{\alpha -1}\rangle }_{AG}e^{-E_{m.ag}/kT}}{{b_{0}(T_{C})}_{AGP}e^{-E_{b.agp}/kT}{\langle m_{B}^{\alpha -1}\rangle }_{AGP}e^{-E_{m.agp}/kT}}$$ (10)

and the strength of the trophic cascade may therefore be expected to decline with a temperature dependence that reflects the temperature dependences of mass and normalized performance for each trophic treatment:

$$ln\;(\frac{M_{B.AGP}}{M_{B.AG}})\propto \frac{\text{ln}\left({b_{0}(T_{C})}_{AG}\right)+ln({\langle m_{B}^{\alpha -1}\rangle }_{AG})-\frac{{E_{b.ag}-E}_{m.ag}}{kT}}{\text{ln}\left({b_{0}(T_{C})}_{AGP}\right)+ln({\langle m_{B}^{\alpha -1}\rangle }_{AGP})-\frac{{E_{b.agp}-E}_{m.agp}}{kT}}$$ (11)

We modeled zooplankton density (N/L) as a function of mean weekly ecosystem temperature T_wj_ and ecosystem trophic structure Z_j_, with ecosystem identity as a random effect.

### Statistical analysis

We tested our hypotheses about whether the effects of temperature on metabolism are modified at the ecosystem level by species interactions using a regression experimental design involving 30 independent ecosystems (Fig 1). We maintained ecosystems at distinct temperatures in a regression design, with mean ecosystem temperatures T_wj_ ranging from 19.7 (±3.15) °C to 26.1 (±3.59) °C (S2 Fig). The regression design allowed us to estimate slopes (e.g., *E*_*R*_, Eq 2) of response variables along a continuous temperature gradient for different trophic structures (A, AG, AGP) by log transforming Eq 2 and fitting linear models to log-transformed response variables along the continuous temperature gradient. We chose the regression design, although unreplicated within temperature levels, because it allowed us to compare activation energies (*E*_*R*_, Eq 2) fitted over a broad range of temperatures; an important test of thermal responses that is not possible with designs with only two or even three temperature levels. Regression designs, even without replication within levels, gain statistical power from the range of x-levels tested [66,67].

We used a mixed effects model (lme function in the nlme package of R) to examine the main and interactive effects of temperature (a continuous fixed factor) and trophic structure (a categorical fixed factor) on net ecosystem oxygen production (NEP), net ecosystem oxygen consumption (ER), and chlorophyll *a* concentration, with a random intercept for individual ecosystems. We used a within-subject mean centering approach to distinguish temperature effects into those associated with an ecosystem’s average temperature (T_j_) over the entire experimental period (a “between-ecosystem” effect) from effects variation in temperature over time (T_wj_) (a within-ecosystem temperature” effect) [68]. The response variable (Y) for each ecosystem *j* in week *w* was modeled as a continuous response to variation in inverted ecosystem temperature (1/kT_wj_) and trophic treatment (*Z*_j_):

ln(Ywj)=β0.j(w)+β1(1kTwj-1kT-j)+β2(1kT-j)+β3(1kTwj-1kT-j)(1kT-j)+β4Zj+β5Zj(1kT-j)+β6Zj(1kT-j-1kT-j)+uj+ewj (12)

where *β*_*0*.*j(i)*_ represents an intercept allowed to vary randomly among ecosystems. The terms in the full model (Eq 12) are the between-ecosystem effect of temperature (*β*_*2*_), estimated as the slope of ln(Y_*wj*_) on the mean temperature over all weeks for ecosystem *j*, expressed as inverse temperature *(1kT-j)*; the within-ecosystem (*β*_*1*_) effect of temperature variation over time, estimated as the slope of ln(Y_*wj*_) versus centered weekly temperature *(1kT-wj)-(1kT-j)*; interaction (*β*_*3*_) between within-ecosystem temporal variation in temperature and the experimental temperature treatment; trophic species interactions (*β*_*4*_); and interactions between species interactions and overall mean (*β*_*5*_) and weekly temperature (*β*_*6*_).

To test our hypothesis that species interactions modify the temperature dependence (*E*_*R*_, Eq 2) of response variables (Y), we compared models with and without trophic-level terms (*β*_*4*_) and interactions between *Z*_*j*_ and temperature (*β*_*5*_, *β*_*6*_). We also tested models without temperature terms for within-system variation (*β*_*4*_). In total, the model set included nine models (Table 1). Response variables were ln transformed prior to analyses to achieve normal distributions, to linearize temperature effects for analysis, and to fit *E*_*R*_ values from Eq 2. When modeling, we centered temperature treatment (1/kT_j_) on the grand mean of all temperatures observations $\overset{-}{T}$ (not shown in Eq 12) to reduce correlations between slope and intercept terms [69].

To test the effect of temperature on trophic cascade strength, we used the following statistical model:

ln(TCij)=β0.p(w)+β1*(1kTwp-1kT-p)+β2*w+β3*(1kTwp-1kT-p)*w+up+ewp, (13)

in which the effect of temperature on trophic cascade strength in each temperature treatment *j* was modeled for each week *w* and for the temperature of the tanks, with random effects *u*_*j*_ assigned for each power treatment (*p*).

We ranked models using Akaike’s Information Criterion weights (using the MuMin package in R), adjusted for small sample sizes (AIC_C_). When two or more models were considered comparable or equivalent (δAIC_C_ < 2), we reported all models meeting this criterion and report averaged coefficients. We estimated the realized temperature dependence of our response variables (slopes) and intercepts for among-ecosystem responses to temperatures by first rearranging Eq 13 to group coefficients by temperature term.

ln(Yjw)=β0.j(w)+(β1+β3*(1kT-j)+β6*Zj)(1kTwj-1kT-j)+(β2+β5*Zj)*(1kT-j)+β4*Zj+uj+ewj. (14)

The pooled coefficients (*β*_2_ + *β*_5_ * *Z*_*j*_) include the temperature dependence of ecosystem responses plus any variation with trophic structure, and give the slope of lines plotted in Figs 2A and 5. The intercept is set by *β*_0.j(*w*)_ + *β*_4_ * *Z*_*j*_, and the remaining coefficient gives within-tank variation as plotted in Fig 6. We estimated confidence intervals for composite terms following [70]. We used R statistical software (R v. 3.5.0 R Developmental Core Team). Our models controlled for the effect of temperature variation over time on ecosystem fluxes and biomass within systems.

We determined the effects of temperature and predator presence on zooplankton abundance data using LMMs with tank as a random effect. We ln transformed data and added 1 to analyze observations of 0 observed zooplankton. In many tanks in which we observed 0 zooplankton in one week, we later observed zooplankton in the same tank. We infer that our observed 0s are most likely failure to observe zooplankton that were in fact present in low densities, so for this reason, we analyzed 0s as ln(1).

## Supporting information

S1 Table Chlorophyll concentration (ln[Chla]) declined over time and varied with trophic treatment. There was a significant temperature * week interaction when we included all the data, from weeks 2−9 (Table S1A). When we used a smaller data set including observations from only weeks 2 to 7, we found evidence for a slight increase in chlorophyll concentration over time (Table S1B). Together, these results suggest that the negative trend in chlorophyll concentration is driven by the drop in week 8 across all treatments. This is concurrent with a cooling event and a large storm.
(DOCX)

S2 Table (A) Zooplankton average body size model selection results. We measured sizes of 641 individual zooplankton. We modeled log(length) of zooplankton in terms of ecosystem weekly temperature (T_wj_), taxon (copepod or *Daphnia*), trophic treatment (AG, AGP), and their interactions. NA indicates that the term was not included in the model. (B) Estimated lengths of *Daphnia* and copepods in treatments with and without predators, from model m2g (Table S2A). AG, algae and grazers; AGP, algae, grazers, and predators; NA, not available.
(DOCX)

S3 Table Phytoplankton species composition and sampling methods. Weekly, we sampled algal assemblages in 100-mL water samples collected from approximately 40 cm below the surface. We used 10-mL subsamples for the identification and counting from each mesocosm. Subsamples were placed in settling chambers and allowed to settle for 24 hours. We counted and identified cells to taxon level using an inverted microscope and the Utermöhl sedimentation method [35]. Phytoplankton density was corrected for volume in each sample using #cells/L = avg cells/field * (F/chamber volume). Cell sizes of phytoplankton taxa were not measured directly; we assigned average cell sizes to each taxon from literature data using the databases www.algaebase.org and www.diatom.org. A water sample 100–300 mL in volume from each mesocosm was filtered onto a 0.2-μm GF/F filter; the water volume varied with the chlorophyll *a* content. Chlorophyll *a* was extracted from the filters in 90% acetone. Chlorophyll *a* concentration, measured in μg/L, was determined fluorometrically using a Trilogy fluorometer (Turner Designs) following Wetzel and Liken (2000).
(DOCX)

S1 Fig Algal community composition in experimental ecosystems shifted over time. Nonmetric multidimensional scaling plot (NMDS) of temporal phytoplankton taxonomic composition for all temperature treatments and trophic levels (Taxa listed in S3 Table). Taxonomic abundances are square root transformed. Each point represents one ecosystem observed at one time, and hotter colors are communities at higher temperatures. NMDS is an iterative search for positions of species, time, temperature, and food chain length on few dimensions (axes) that minimizes departure from monotonicity in the association between distance (dissimilarity) in the original data and ordination space. See S3 Fig for comparisons of phytoplankton taxonomic composition versus temperature. NMDS, nonmetric multidimensional scaling plot.
(JPG)

S2 Fig Temperatures from data loggers in ecosystems illustrate a cooling trend over the course of the experiment, and variable temperatures from day to day. Differences among ecosystems were maintained by heaters of different power (watts). Red colors indicate warmer ecosystems at higher wattage and blue colors indicate cooler ecosystems.
(JPG)

S3 Fig Temperature did not clearly shift algal community composition in experimental ecosystems. Nonmetric multidimensional scaling plot (NMDS) of temporal phytoplankton taxonomic composition for all temperature treatments and trophic levels (taxa listed in S1 Table). Taxonomic abundances are square root transformed. Each point represents one ecosystem observed at one time, and lighter colors are communities at higher temperatures. NMDS is an iterative search for positions of species, time, temperature, and food chain length on few dimensions (axes) that minimizes departure from monotonicity in the association between distance (dissimilarity) in the original data and ordination space. See S2 Fig for comparisons of phytoplankton taxonomic composition versus week. NMDS, nonmetric multidimensional scaling plot.
(JPG)

S4 Fig Phytoplankton abundance, estimated as ln[chlorophyll a], for each observation date over the course of the experiment. Lines connect observations from the same ecosystem. Trophic treatments are separated: A, algae only; AG, algae + grazers; and AGP, algae + grazers + predators.
(JPG)

## Abbreviations

A: algae only
AG: algae and grazers
AGP: algae, grazers, and predators
DO: dissolved oxygen
ER: ecosystem respiration
LMM: linear mixed effects model
MTE: metabolic theory of ecology
NEP: net ecosystem production
TPC: thermal performance curve
