# Supplementary material for: Trophic interactions modify the temperature dependence of community biomass and ecosystem function
Source: PLoS Biol. 2019 Jun 10;17(6):e2006806. doi: 10.1371/journal.pbio.2006806 (PMC6586427; doi:10.1371/journal.pbio.2006806)
Supplement: S1 Table — There was a significant temperature * week interaction when we included all the data, from weeks 2−9 (Table S1A). When we used a smaller data set including observations from only weeks 2 to 7, we found evidence for a slight increase in chlorophyll concentration over time (Table S1B). Together, these results suggest that the negative trend in chlorophyll concentration is driven by the drop in week 8 across all treatments. This is concurrent with a cooling event and a large storm. (DOCX) [file pbio.2006806.s001.docx]

S1_Table

Table S1A: Model selection results: Chlorophyll a variation over time and with trophic treatment, weeks 2-9.

| Int TrophicLev | Week | TL*Wk | df | logLik | AICc | d | w |
| --- | --- | --- | --- | --- | --- | --- | --- |
| CT1 2.54 + | -0.12 | + | 8 | -194.72 | 406.06 | 0.00 | 9.224069e-01 |
| CT2 2.82 + | -0.17 | NA | 6 | -199.33 | 411.02 | 4.97 | 7.698761e-02 |
| CT3 2.41 NA | -0.17 | NA | 4 | -206.27 | 420.71 | 14.66 | 6.054409e-04 |
| CT4 1.49 NA | NA | NA | 3 | -264.15 | 534.41 | 128.35 | 1.240275e-28 |

Table S1B: Model selection results: Chlorophyll a variation over time and with trophic treatment, weeks 2-7.

| Int TrophicLev | Week | TL*Wk | df | logLik | AICc | d | w |
| --- | --- | --- | --- | --- | --- | --- | --- |
| CT1s 1.91 + | 0.05 | + | 8 | -83.30 | 183.44 | 0.00 | 1.000000e+00 |
| CT2s 2.28 + | -0.03 | NA | 6 | -108.23 | 228.94 | 45.50 | 1.319628e-10 |
| CT3s 1.90 NA | -0.03 | NA | 4 | -117.12 | 242.47 | 59.03 | 1.517267e-13 |
| CT4s 1.76 NA | NA | NA | 3 | -119.11 | 244.36 | 60.92 | 5.905315e-14 |
