# Supplementary material for: Trophic interactions modify the temperature dependence of community biomass and ecosystem function
Source: PLoS Biol. 2019 Jun 10;17(6):e2006806. doi: 10.1371/journal.pbio.2006806 (PMC6586427; doi:10.1371/journal.pbio.2006806)
Supplement: S2 Table — (A) Zooplankton average body size model selection results. We measured sizes of 641 individual zooplankton. We modeled log(length) of zooplankton in terms of ecosystem weekly temperature (Twj), taxon (copepod or Daphnia), trophic treatment (AG, AGP), and their interactions. NA indicates that the term was not included in the model. (B) Estimated lengths of Daphnia and copepods in treatments with and without predators, from model m2g (Table S2A). AG, algae and grazers; AGP, algae, grazers, and predators; NA, not available. (DOCX) [file pbio.2006806.s002.docx]

### **S2_Table. Zooplankton average body size model selection results**. We measured sizes of 641 individual zooplankton. We modeled log(length) of zooplankton in terms of ecosystem weekly temperature (T_wj_), taxon (copepod or *Daphnia*), trophic treatment (AG, AGP) and their interactions. NA indicates that the term was not included in the model.

Table S2A


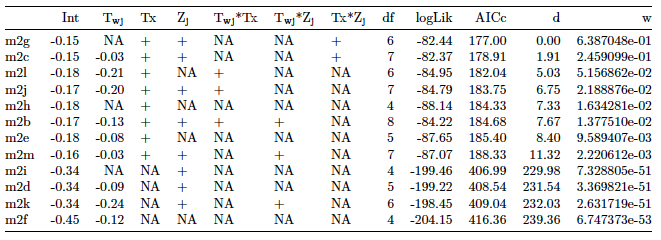


Table S2B. Estimated lengths of Daphnia and copepods in treatments with and without predators, from model m2g (Table S2A).

**
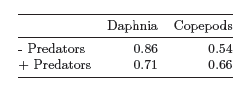
**
