# Supplementary material for: Trophic interactions modify the temperature dependence of community biomass and ecosystem function
Source: PLoS Biol. 2019 Jun 10;17(6):e2006806. doi: 10.1371/journal.pbio.2006806 (PMC6586427; doi:10.1371/journal.pbio.2006806)
Supplement: S3 Table — Weekly, we sampled algal assemblages in 100-mL water samples collected from approximately 40 cm below the surface. We used 10-mL subsamples for the identification and counting from each mesocosm. Subsamples were placed in settling chambers and allowed to settle for 24 hours. We counted and identified cells to taxon level using an inverted microscope and the Utermöhl sedimentation method [35]. Phytoplankton density was corrected for volume in each sample using #cells/L = avg cells/field * (F/chamber volume). Cell sizes of phytoplankton taxa were not measured directly; we assigned average cell sizes to each taxon from literature data using the databases www.algaebase.org and www.diatom.org. A water sample 100–300 mL in volume from each mesocosm was filtered onto a 0.2-μm GF/F filter; the water volume varied with the chlorophyll a content. Chlorophyll a was extracted from the filters in 90% acetone. Chlorophyll a concentration, measured in μg/L, was determined fluorometrically using a Trilogy fluorometer (Turner Designs) following Wetzel and Liken (2000). (DOCX) [file pbio.2006806.s003.docx]

**S3_Table. Phytoplankton species composition and sampling methods.**

Weekly, we sampled phytoplankton, chlorophyll *a*, zooplankton, and oxygen concentrations. We sampled algal assemblages in 100-mL water samples collected from ~40-cm below the surface. We used 10mL subsamples for the identification and counting from each mesocosm. Subsamples were placed in settling chambers and allowed to settle for 24h. We counted and identified cells to taxon level using an inverted microscope and the Utermöhl sedimentation method [35] . Phytoplankton density was corrected for volume in each sample using:

#cells/L = avg cells/field * (F/chamber volume)

Cell sizes of phytoplankton taxa were not measured directly, we assigned average cell sizes to each taxon from literature data using the databases [www.algaebase.](http://www.algaebase.)org and [www.diatom.org](http://www.diatom.org).

100 to 300mL volume of water samples from each mesocosm was filtered onto a 0.2μm GF/F filter; the water volume varied with the chlorophyll *a* content. Chlorophyll *a* was extracted from the filters in 90% acetone. Chlorophyll *a* concentration, measured in μg/L, was determined fluorometrically using a Trilogy fluorometer (Turner Designs) following Wetzel and Liken (2000).

| Taxon | Group |
| --- | --- |
| Amoeboid | Amoeboid |
| Gloeocystis | Chlorophyta |
| Arthrodesmus | Chlorophyta |
| Chlamydomonas | Chlorophyta |
| Paramecium | Ciliate |
| Chrysosphaerella | Cryptophyte |
| Dinobryon | Cryptophyte |
| Fragilaria | Cryptophyte |
| Fragilaria | Cryptophyte |
| Chrysophyte | Cryptophyte |
| Microcystis | Cyanobacteria |
| Oscillatoria | Cyanobacteria |
| Anabaena | Cyanobacteria |
| Coelosphaerium | Cyanobacteria |
| Planktothrix | Cyanobacteria |
| Merismopedia | Cyanobacteria |
| Chroococcus | Cyanobacteria |
| Synechococcus | Cyanobacteria |
| Merismopedia | Cyanobacteria |
| Fischerella | Cyanobacteria |
| Tabellaria | Diatom |
| Eunotia | Diatom |
| Melosira | Diatom |
| Diatom3 | Diatom |
| Gymnodinium | Dinoflagelate |
