# Supplementary figures and images for: Trophic interactions modify the temperature dependence of community biomass and ecosystem function
Source: PLoS Biol. 2019 Jun 10;17(6):e2006806. doi: 10.1371/journal.pbio.2006806 (PMC6586427; doi:10.1371/journal.pbio.2006806)

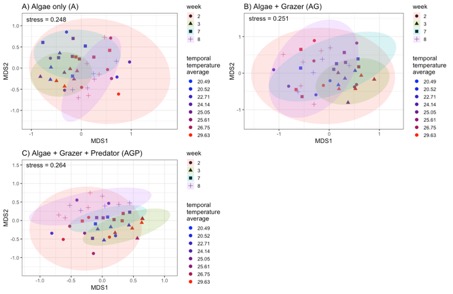

Supplement: S1 Fig — Nonmetric multidimensional scaling plot (NMDS) of temporal phytoplankton taxonomic composition for all temperature treatments and trophic levels (Taxa listed in S3 Table). Taxonomic abundances are square root transformed. Each point represents one ecosystem observed at one time, and hotter colors are communities at higher temperatures. NMDS is an iterative search for positions of species, time, temperature, and food chain length on few dimensions (axes) that minimizes departure from monotonicity in the association between distance (dissimilarity) in the original data and ordination space. See S3 Fig for comparisons of phytoplankton taxonomic composition versus temperature. NMDS, nonmetric multidimensional scaling plot. (JPG) [file pbio.2006806.s004.jpg]

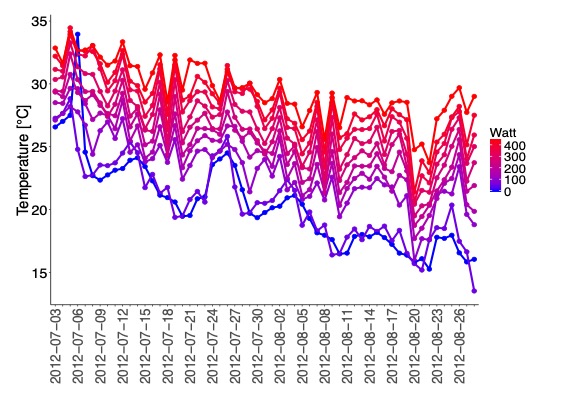

Supplement: S2 Fig — Differences among ecosystems were maintained by heaters of different power (watts). Red colors indicate warmer ecosystems at higher wattage and blue colors indicate cooler ecosystems. (JPG) [file pbio.2006806.s005.jpg]

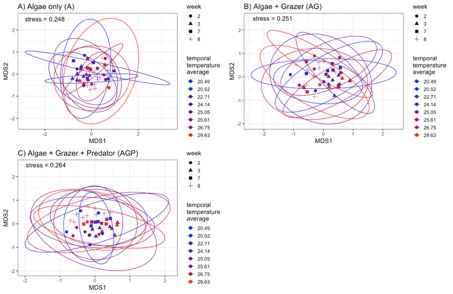

Supplement: S3 Fig — Nonmetric multidimensional scaling plot (NMDS) of temporal phytoplankton taxonomic composition for all temperature treatments and trophic levels (taxa listed in S1 Table). Taxonomic abundances are square root transformed. Each point represents one ecosystem observed at one time, and lighter colors are communities at higher temperatures. NMDS is an iterative search for positions of species, time, temperature, and food chain length on few dimensions (axes) that minimizes departure from monotonicity in the association between distance (dissimilarity) in the original data and ordination space. See S2 Fig for comparisons of phytoplankton taxonomic composition versus week. NMDS, nonmetric multidimensional scaling plot. (JPG) [file pbio.2006806.s006.jpg]

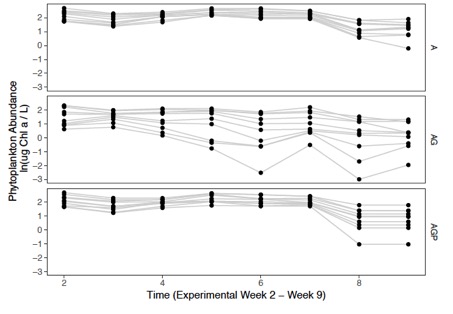

Supplement: S4 Fig — Lines connect observations from the same ecosystem. Trophic treatments are separated: A, algae only; AG, algae + grazers; and AGP, algae + grazers + predators. (JPG) [file pbio.2006806.s007.jpg]
